# Activation of STING requires palmitoylation at the Golgi

**DOI:** 10.1038/ncomms11932

**Published:** 2016-06-21

**Authors:** Kojiro Mukai, Hiroyasu Konno, Tatsuya Akiba, Takefumi Uemura, Satoshi Waguri, Toshihide Kobayashi, Glen N. Barber, Hiroyuki Arai, Tomohiko Taguchi

**Affiliations:** 1 Department of Health Chemistry, Graduate School of Pharmaceutical Sciences, University of Tokyo, 7-3-1, Hongo, Bunkyo-ku, Tokyo 113-0033, Japan; 2 Lipid Biology Laboratory, RIKEN, 2-1, Hirosawa, Wako-shi, Saitama 351-0198, Japan; 3 Department of Cell Biology and Sylvester Comprehensive Cancer Center, University of Miami School of Medicine, Miami, Florida 33136, USA; 4 Department of Anatomy and Histology, Fukushima Medical University School of Medicine, Hikarigaoka, Fukushima 960-1295, Japan; 5 Pathological Cell Biology Laboratory, Graduate School of Pharmaceutical Sciences, University of Tokyo, Tokyo 113-0033, Japan

## Abstract

Stimulator of interferon genes (STING) is essential for the type I interferon response against DNA pathogens. In response to the presence of DNA and/or cyclic dinucleotides, STING translocates from the endoplasmic reticulum to perinuclear compartments. However, the role of this subcellular translocation remains poorly defined. Here we show that palmitoylation of STING at the Golgi is essential for activation of STING. Treatment with palmitoylation inhibitor 2-bromopalmitate (2-BP) suppresses palmitoylation of STING and abolishes the type I interferon response. Mutation of two membrane-proximal Cys residues (Cys88/91) suppresses palmitoylation, and this STING mutant cannot induce STING-dependent host defense genes. STING variants that constitutively induce the type I interferon response were found in patients with autoimmune diseases. The response elicited by these STING variants is effectively inhibited by 2-BP or an introduction of Cys88/91Ser mutation. Our results may lead to new treatments for cytosolic DNA-triggered autoinflammatory diseases.

## Abstract (web summary)

STING is essential for the type I interferon immune response to foreign DNA. Here, the authors show that palmitoylation of STING at the Golgi is required for activating downstream signalling, and increased Golgi localization of certain STING variants may cause autoimmune disease in some cases.

## Graphical abstract

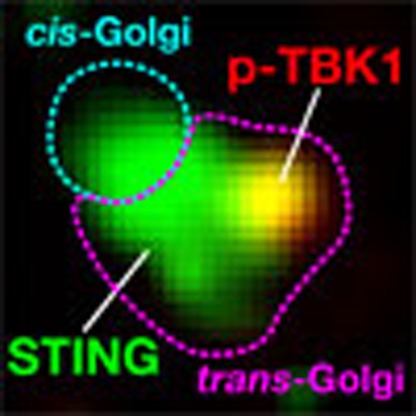

The innate immune response is critical for efficient host defense against microbial invasion. Invading pathogens are identified by pattern recognition receptors in the host cell, which initiates a series of signalling events that leads to the production of type I interferons, proinflammatory cytokines and other downstream antiviral proteins^1^,^2^. The pattern recognition receptors include Toll-like receptors, RIG-I-like receptors and nucleotide-binding domain and leucine-rich repeat-containing receptors that sense microbial molecules such as CpG DNA, viral RNAs and lipopolysaccharides^3^,^4^,^5^. Furthermore, an endoplasmic reticulum (ER)-associated molecule referred to as stimulator of interferon genes (STING, also known as MITA, ERIS, MPYS or TMEM173)^6^,^7^,^8^,^9^,^10^ has recently been shown to control a new sensing pathway that is essential for detecting cytosolic DNA or cyclic dinucleotides (CDNs) which include cyclic GMP–AMP (cGAMP)^11^,^12^,^13^.

Besides the essential roles of STING in protecting the host against DNA pathogens, STING is also involved in the pathogenesis of autoinflammation caused by self-DNA in murine models (*DnaseII*^*−/−*^ and *DnaseIII (Trex1)*^*−/−*^)^14^,^15^,^16^. This suggests that STING may also be involved in human autoinflammatory disorders such as Aicardi–Goutieres syndrome and systemic lupus erythematosus that are associated with mutations of human DNase III^17^,^18^. Furthermore, mutations in STING are found in patients with an autoinflammatory disease called STING-associated vasculopathy with onset in infancy (SAVI)^19^ and more recently in patients with lupus-like syndromes^20^. The STING variants found in SAVI patients appear to be constitutively activated without CDNs and interestingly do not localize to the ER^20^,^21^.

After DNA and/or CDN binding, STING translocates from the ER to perinuclear compartments that include the Golgi, endosomes and autophagy-related compartments^6^,^22^. Treatment with brefeldin A (BFA) or expression of *Shigella* effector IpaJ, which blocks ER-to-Golgi traffic, abolishes the STING-dependent signalling events that include phosphorylation of TANK-binding kinase 1 (TBK1) and the transcription factor interferon regulatory factor 3 (IRF3) and induction of interferon β (IFNβ)^6^,^21^,^23^. These results, together with the observation that SAVI–STINGs (activated without CDNs) do not localize to the ER, all indicate the contribution of post-ER compartments to the activation of STING. However, the molecular mechanism underlying the activation of STING in post-ER compartments is not understood.

In the present study, we showed that STING is palmitoylated at the Golgi and this post-translational modification is required for the activation of STING-dependent downstream signalling for the type I interferon response.

## Results

### Post-ER trafficking route of STING after stimulation

**Figure 1:**
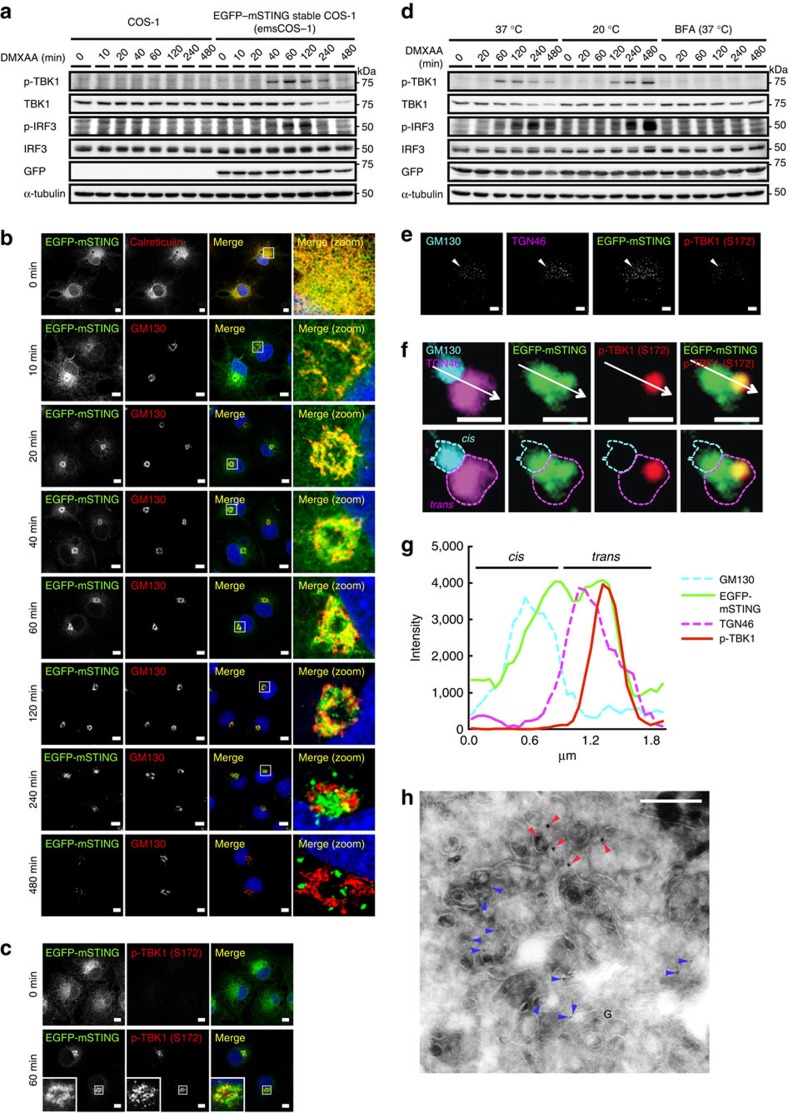
STING activates TBK1 at the Golgi. (**a**) Western blots of cell lysates of emsCOS-1 cells stimulated with DMXAA for the indicated times. (**b**) Cells were stimulated with DMXAA for the indicated times, fixed, permeabilized and stained for calreticulin (an ER protein) or GM130 (a Golgi protein). Nuclei were stained with DAPI (blue). Scale bars, 10 μm. (**c**) Immunostaining of phosphorylated TBK1 after the treatment with DMXAA (0 or 1 h). Scale bars, 10 μm. (**d**) The effect of low temperature (20 °C) or BFA on phosphorylation of TBK1 and IRF3. (**e**) Immunostaining of phosphorylated TBK1 (red) in cells treated with nocodazole and DMXAA. GM130 (a CGN protein, cyan) and TGN46 (a TGN protein, magenta) were co-stained. Scale bars, 10 μm. (**f**) The mini-Golgi indicated by arrow (**e**) was magnified. The *cis* and *trans*-regions of the mini-Golgi were outlined in the images at the bottom row. Scale bars, 1 μm. (**g**) Fluorescence intensity profile along the arrow (**f**) is shown. (**h**) emsCOS-1 cells were stimulated with DMXAA for 1 h, fixed and processed for ultrathin-cryosections. They were immunostained with anti-p-TBK1 (rabbit) and anti-TGN46 (sheep) antibodies. As secondary antibodies, colloidal gold particle-conjugated donkey anti-rabbit antibody (12 nm) and donkey anti-sheep antibody (6 nm) were used. Red and blue arrowheads indicate p-TBK1 and TGN46 labelling, respectively. G, the Golgi stack. Scale bars, 200 nm.

To examine the intracellular behaviour of STING, we added an amino-terminal EGFP tag to mouse STING (EGFP–mSTING) and used DMXAA, a membrane-permeable mouse-specific STING agonist. When EGFP–mSTING was transiently expressed in HEK293T cells that lack endogenous STING^12^, it activated the IRF3 promoter in a DMXAA-dependent manner (Supplementary Fig. 1a), indicating that tagging EGFP to mSTING did not impair the STING activity. EGFP–mSTING was then stably expressed in COS-1 cells (emsCOS-1 cells), a cell line with distinctly separate organelles^24^. emsCOS-1 cells induced downstream genes of STING, such as *IFNB*, *IL8* and *TNF* on DMXAA stimulation (Supplementary Fig. 1b). Following the binding of DMXAA or CDNs, STING recruits TBK1 to induce autophosphorylation of TBK1 (ref. 23). Phosphorylated TBK1 then phosphorylates transcription factor IRF3 to stimulate transcription of *IFNB*^25^. Phosphorylation of TBK1 peaked at 60 min after DMXAA stimulation in emsCOS-1 cells (Fig. 1a). Phosphorylated IRF3 was observed for 60–120 min (Fig. 1a). As was shown previously for endogenous STING^23^, EGFP–mSTING localized at the ER in unstimulated cells and exited the ER after stimulation (Fig. 1b). Co-immunostaining with GM130, a Golgi protein, showed that EGFP–mSTING mostly localized to the Golgi at 60 min (Fig. 1b). Phosphorylated TBK1 was partly co-localized with EGFP–mSTING at this time (Fig. 1c). EGFP–mSTING co-localized with rab11, a recycling endosomal protein at 240 min, and with p62, a lysosomal/autophagic protein at 480 min (Supplementary Fig. 1c). These results suggest that STING sequentially moved from the ER to the Golgi to recycling endosomes and then to the lysosomal/autophagic membranes after stimulation and that phosphorylation of TBK1, a hallmark of STING activation, occurred at the Golgi.

### STING activates TBK1 at the TGN

We examined whether inhibition of membrane trafficking affected the phosphorylation of TBK1. Treatment with BFA, which blocks ER-to-Golgi traffic^26^ (Supplementary Fig. 2a), suppressed phosphorylation of TBK1 and IRF3 (Fig. 1d)^6^,^23^. Low temperature (20 °C) impairs membrane traffic from the Golgi, but not from the ER to the Golgi^27^. EGFP–mSTING still localized to the Golgi 480 min after DMXAA stimulation at 20 °C (Supplementary Fig. 2b), while EGFP–mSTING localized to the lysosomal/autophagic membranes at that time at 37 °C (Supplementary Fig. 1c). Under the low temperature condition, DMXAA-induced phosphorylation of TBK1 and IRF3 even more strongly than at 37 °C (Fig. 1d). These data suggested that the Golgi was the organelle where STING activated TBK1 and IRF3.

The Golgi is a polarized organelle^28^ that has distinct functional domains, such as the *cis*-Golgi network (CGN) and *trans*-Golgi network (TGN). Treatment of cells with the microtubule-depolymerizing agent nocodazole results in dispersed Golgi stacks in the cytoplasm, and this fragmentation facilitates the analysis of *cis*-to-*trans* polarity of the Golgi^29^. With this method, we found that, while STING localized at both the CGN and TGN, phosphorylated TBK1 was confined to a subdomain of the TGN (Fig. 1e–g and Supplementary Fig. 3). We also found that phosphorylated TBK1 was confined to a subdomain of the TGN in mouse embryonic fibroblasts (MEFs) (Supplementary Fig. 4). Immunoelectron microscopy of emsCOS-1 cells 60 min after DMXAA stimulation showed that phosphorylated TBK1 was mostly localized to tubular or vesicular profiles adjacent to the TGN46-positive structures in the TGN region (Fig. 1h).

### STING palmitoylation is required for the type I IFN response

**Figure 2:**
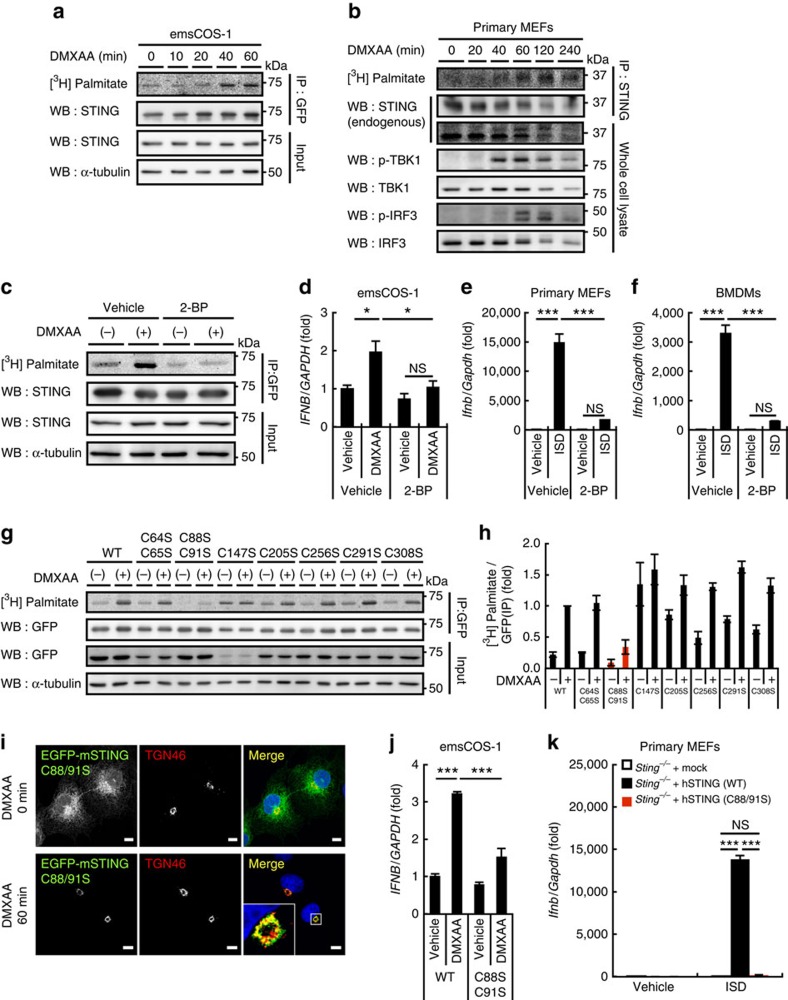
Palmitoylation at Cys88/91 of STING is required for the type I interferon response. (**a**) emsCOS-1 cells were starved for 1 h, followed by the incubation with [^3^H] palmitate for 1 h. Cells were then stimulated with DMXAA for the indicated times. Cell lysates were prepared and EGFP–STING was immunoprecipitated with anti-GFP antibody. Cell lysates and the immunoprecipitates were analysed by western blot and autoradiography. (**b**) Primary MEFs were examined as in **a**. For immunoprecipitation, anti-STING antibody was used. (**c**) The effect of 50 μM 2-BP on the palmitoylation of STING in emsCOS-1 cells. (**d**) Quantitative real-time PCR (qRT-PCR) of the expression of IFNβ in emsCOS-1 cells that were pretreated with vehicle or 50 μM 2-BP for 1 h and then stimulated with DMXAA for 12 h. (**e**,**f**) qRT-PCR of the expression of IFNβ in primary MEFs (**e**) or BMDMs (**f**) that were pretreated with vehicle or 50 μM 2-BP for 1 h and then stimulated with ISD for 3 h. (**g**) COS-1 cells that stably express EGFP-mouse STING with indicated Cys mutations were metabolically labelled, stimulated with DMXAA, and analysed by western blot and autoradiography. (**h**) The band intensities in **g** were quantified and [^3^H palmitate]/[GFP] were calculated. The data are normalized to the value of WT-STING with DMXAA treatment and represent mean±s.e.m. of two independent experiments. (**i**) COS-1 cells that stably express STING (C88/91S) were stimulated with DMXAA for 1 h, fixed, permeabilized and stained for TGN46 (red). Nuclei were stained with DAPI (blue). Scale bars, 10 μm. (**j**) COS-1 cells that stably express STING (WT or C88/91S) were stimulated with DMXAA for 12 h. qRT-PCR of the expression of IFNβ was then performed. (**k**) Primary *Sting*^*−/−*^ MEFs were reconstituted with human STING variants using retroviruses. The cells were stimulated with ISD for 6 h, and qRT-PCR of the expression of IFNβ was performed. Data in **d**,**e**,**f**,**j** and **k** are mean±s.e.m. from three independent experiments. *P<0.01, ***P<0.001, NS, not significant (one-way analysis of variance).

We hypothesized that STING is post-translationally modified at the TGN to activate downstream signalling and found that palmitoylation of STING was required for the type I interferon response. Using a [^3^H] palmitate metabolic labelling approach, we found that [^3^H] labelling of STING was greatly increased after DMXAA stimulation (Fig. 2a). The timing of the labelling of STING correlated well with the timing of the phosphorylation of TBK1 in emsCOS-1 cells (Fig. 1a). STING localized to the Golgi 40–60 min after DMXAA stimulation of emsCOS-1 cells (Fig. 1b). These results suggested that palmitoylation of STING occurred at the Golgi and participated in the type I interferon response. Although STING already localized to the Golgi 20 min after the stimulation (Fig. 1b), at that time [^3^H] labelling of STING was as weak as before the stimulation, suggesting that palmitoylation of STING occurred in later Golgi compartments that include the TGN. We also found the stimulation-dependent [^3^H] labelling of STING in MEFs (Fig. 2b and Supplementary Fig. 5a). The timing of [^3^H] labelling of STING was correlated with that of phosphorylation of TBK1 and IRF3 (Fig. 2b) and that of the Golgi localization of STING (Supplementary Fig. 6). The palmitoylation of STING lingered even after STING was transported to the degradation compartments (for example, 240 min after DMXAA stimulation in Fig. 2b), suggesting that depalmitoylation of STING did not occur during its transport from the Golgi to the degradation compartments.

Treating emsCOS-1 cells with the palmitoylation inhibitor 2-BP effectively abolished the DMXAA-induced incorporation of [^3^H] into STING (Fig. 2c), confirming the palmitoylation of STING. More importantly, 2-BP suppressed DMXAA-, or interferon-stimulatory DNA (ISD)-triggered induction of downstream genes, such as *IFNB*, *IL8*, *IL6* and *TNF* in emsCOS-1 cells, primary MEFs and bone marrow-derived macrophages (BMDMs) (Fig. 2d–f and Supplementary Fig. 7). The effect of 2-BP was selective for STING-mediated cytosolic DNA sensing over other innate immune signalling, since we did not observe drastic effects of 2-BP on the Toll-like receptor 3 pathway for the detection of extracellular/luminal double-stranded RNAs or on the RIG-I-like receptor (RLR) pathway for the detection of cytosolic RNAs (Supplementary Fig. 8). 2-BP did not impair the translocation of STING from the ER to the Golgi (Supplementary Fig. 9a), suggesting that palmitoylation may not be required for the binding of DMXAA to STING. 2-BP also did not impair the post-Golgi trafficking of STING from the Golgi to lysosomal/autophagic membranes (Supplementary Fig. 9a) or the degradation of STING (Supplementary Fig. 10a,b).

Cysteine (Cys) residues are the site of protein palmitoylation^30^. Mammalian STINGs have several conserved Cys residues that may be localized in the cytoplasmic region or near the end of the transmembrane region^31^ (Supplementary Fig. 11). We generated STING mutants with single Cys to Ser substitutions (Cys147, 205, 256, 291 and 308) or dual substitutions (Cys64/65 and Cys88/91) for adjacent or proximal Cys residues, and found that the Cys88/91 dual mutation resulted in the drastic loss of palmitoylation (Fig. 2g,h). Single mutations of Cys88 or Cys91 also reduced [^3^H] labelling, but to a lesser extent (Supplementary Fig. 12). STING (C88/91S), like the wild type (WT), moved from the ER to the Golgi after DMXAA stimulation (Fig. 2i), but could not induce DMXAA-triggered downstream genes (Fig. 2j and Supplementary Fig. 13a,b). The post-Golgi trafficking and the degradation of STING (C88/91S) were essentially similar to those of STING (WT) (Supplementary Figs 9b and 10c). These data suggested that palmitoylation at Cys88/91 was critical for the type I interferon response, but not for the trafficking of STING. Although the Cys88/91 dual mutation resulted in the significant loss of palmitoylation, some labelling was still detected after DMXAA stimulation. STING (WT and C88/91S) was treated with hydoxylamine, a treatment that cleaves palmitoyl–thioester bonds^32^ (Supplementary Fig. 5b). The treatment of stimulated STING (WT) and STING (C88/91S) mostly reduced the [^3^H] labelling. These results indicated that in addition to palmitoylation at Cys88/91, stimulated STING in emsCOS-1 cells undergoes palmitoylation at Cys residue (s) other than Cys88/91. In the case of STING that was reconstituted into MEFs, the [^3^H] labelling of STING also diminished after the hydroxylamine treatment (Supplementary Fig. 5a). STING (C88/91S) in MEFs could not induce ISD-triggered downstream genes (Fig. 2k and Supplementary Fig. 13c–e), thus further supporting the role of palmitoylation at Cys88/91 of STING in the type I interferon response.

We also examined whether the C88/91S mutant activated the type I interferon response that is elicited by cGAMP, an endogenous STING ligand^11^. STING, when expressed at low levels in HEK293T cells, showed CDNs-dependent type I interferon response^12^. With this method, we confirmed that human STING (WT) could activate IRF3-, IFNβ- or NF-κB-promoters (Supplementary Fig. 13f–h), induce *IFNB* and *IFIT1* (Supplementary Fig. 13i–j), and promote phosphorylation of IRF3 following cGAMP stimulation (Supplementary Fig. 13k). In contrast, human STING (C88/91S) could not activate these same type I interferon responses (Supplementary Fig. 13f–k), suggesting that palmitoylation was also essential for cGAMP-induced STING activation.

**Figure 3:**
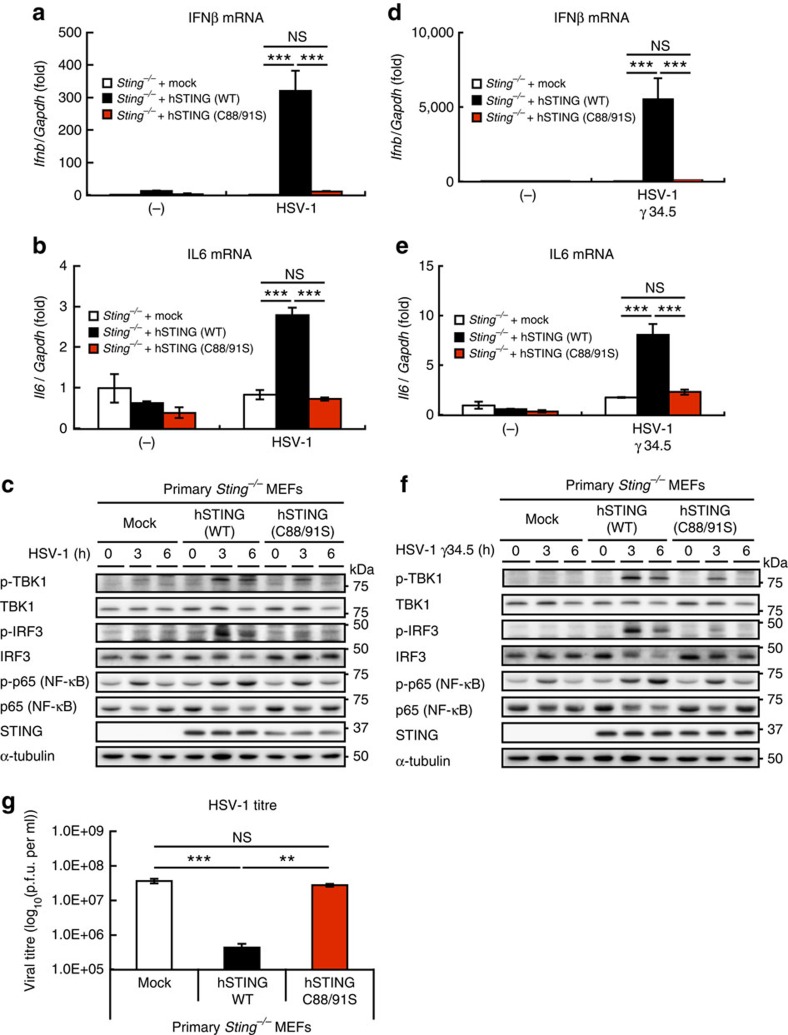
STING C88/91S mutant is unable to induce the type I interferon response against HSV-1 infection. (**a**,**b**) Primary *Sting*^*−/−*^ MEF cells were reconstituted with human STING variants using retroviruses. The cells were infected with HSV-1 (multiplicity of infection (MOI)=10) for 3 h, and qRT-PCR was performed (**a**, IFNβ mRNA; **b**, IL6 mRNA). (**c**) Cell lysates were prepared the indicated times after HSV-1 infection, and analysed by western blot. (**d**–**f**) Cellular response to HSV-1 γ34.5 was analysed as (**a**–**c**). (**g**) Reconstituted primary *Sting*^*−/−*^ MEF cells with human STING (WT or C88/91) were infected with HSV-1 (MOI=1) for 24 h, and then plaque assay was performed. Data in **a**,**b**,**d**,**e** and **g** are mean±s.e.m. from three independent experiments. **P<0.005, ***P<0.001, NS, not significant (one-way analysis of variance).

The C88/91S mutant was used to determine whether palmitoylation is also needed in the host defense response against DNA viruses^6^. Reconstituting *Sting*^*−/−*^ MEFs with human STING (WT) resulted in the induction of *Ifnb* and *Il6*, and phosphorylation of TBK1, IRF3 and p65 (NF-κB) on infection with herpes simplex virus-1 (HSV-1), a double-stranded DNA virus (Fig. 3a–c), whereas reconstituting the MEFs with human STING (C88/91S) failed to activate the type I interferon response. STING (C88/91S) was also unable to stimulate the type I interferon response following infection with HSV-1 γ34.5, a HSV-1 variant (Fig. 3d–f). Significantly, human STING (WT) suppressed HSV-1 replication, whereas human STING (C88/91S) could not (Fig. 3g), suggesting that the palmitoylation of STING is essential for virus resistance.

### SAVI–STINGs require palmitoylation for their activity

**Figure 4:**
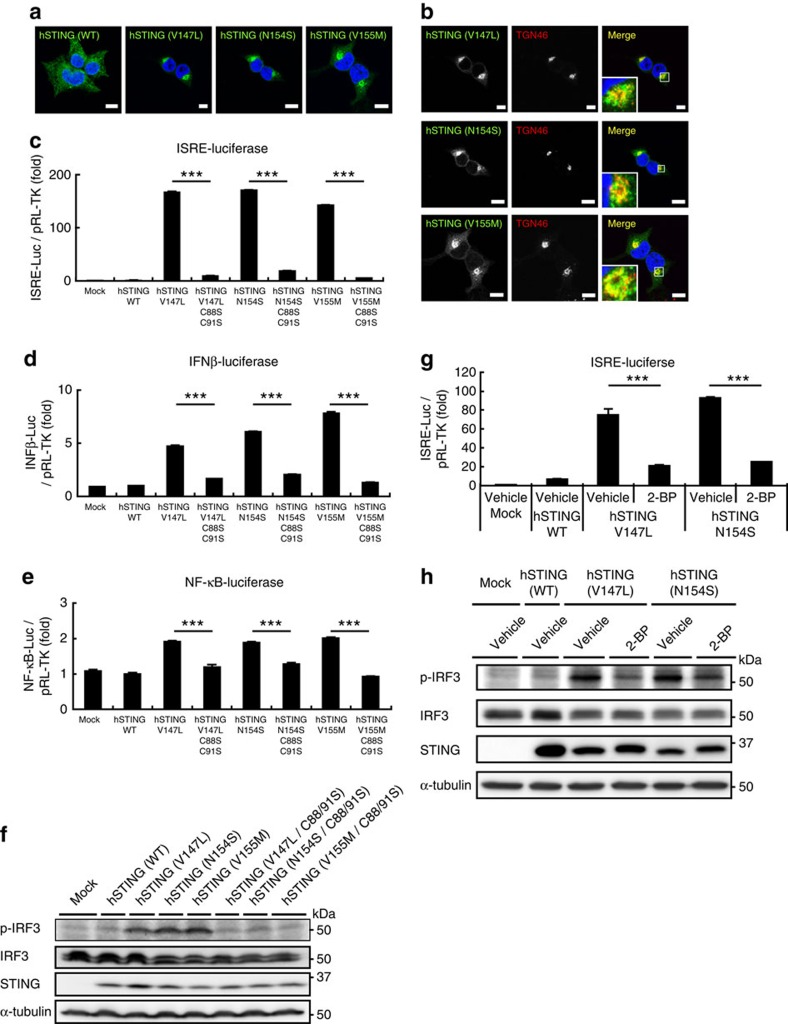
Suppression of STING palmitoylation inhibits the type I interferon response elicited by the mutant STING variants associated with SAVI. (**a**) HEK293T cells were transfected as indicated, fixed, permeabilized and stained with anti-STING antibody. (**b**) Co-immunostaining of cells in **a** with anti-TGN46 (a TGN protein). Nuclei were stained with DAPI (blue). Scale bars, 10 μm. (**c**–**e**) HEK293T cells were transfected as indicated, together with an ISRE (also known as PRDIII or IRF-E)-luciferase reporter (**c**), IFNβ-luciferase reporter (**d**) and NF-κB-luciferase reporter (**e**) for 24 h. Luciferase activity was then measured. (**f**) Cells were transfected as indicated. Cell lysates were prepared 24 h after transfection, and analysed by western blot. (**g**) HEK293T cells that stably express SAVI–STING (V147L or N154S) were transfected with an ISRE-luciferase reporter for 24 h. Luciferase activity was then measured. 2-BP (50 μM) was added 6 h after the transfection. (**h**) Cells that stably express SAVI–STINGs were treated with 50 μM 2-BP for 24 h. Cell lysates were then prepared, and analysed by western blot. Data in **c**,**d**,**e** and **g** are mean±s.e.m. from three independent experiments. ***P<0.001 (one-way analysis of variance).

In 2014, mutations in human STING were found in patients with early-onset systemic inflammation, cutaneous vasculopathy and pulmonary inflammation^19^,^20^. The disease was named SAVI. SAVI patients have a point mutation in exon 5 of STING (V147L, N154S or V155M). Such STING variants exhibit a gain-of-function phenotype and are able to stimulate the production of an IFNβ reporter construct irrespective of the presence of cGAMP^19^,^20^. When transiently expressed in HEK293T cells, all the SAVI–STINGs localized to perinuclear compartments, but not to the ER (Fig. 4a), which is consistent with previous observations^20^,^21^. We further found that some portion of SAVI–STINGs localized to the TGN (Fig. 4b), where STING was activated (Fig. 1e–h). We then examined whether the palmitoylation was also required for the activity of the SAVI–STINGs. The three SAVI–STINGs (V147L, N154S and V155M), when transiently expressed in HEK293T cells, resulted in activation of the IRF3-, IFNβ- and NF-κB-promoters (Fig. 4c–e), and the phosphorylation of IRF3 (Fig. 4f). In contrast, SAVI–STINGs with the C88/91S mutation could not activate the IRF3-, IFNβ- or NF-κB-promoters (Fig. 4c–e), and could not induce the phosphorylation of IRF3 (Fig. 4f). We also examined effects of 2-BP on the SAVI–STING-induced type I interferon response. HEK293T cells that stably express SAVI–STING (V147L and N154S) showed the type I interferon responses under unstimulated conditions. Treatment of the cells with 2-BP significantly reduced the type I interferon responses, such as induction of the IRF3 promoter (Fig. 4g) and phosphorylation of IRF3 (Fig. 4h). Thus, inhibition of palmitoylation could suppress a gain-of-function phenotype in the SAVI–STING. Effects of 2-BP was not determined on V155M variant, because stable cell lines could not be generated.

### Disturbing Golgi lipid order suppresses STING activation

**Figure 5:**
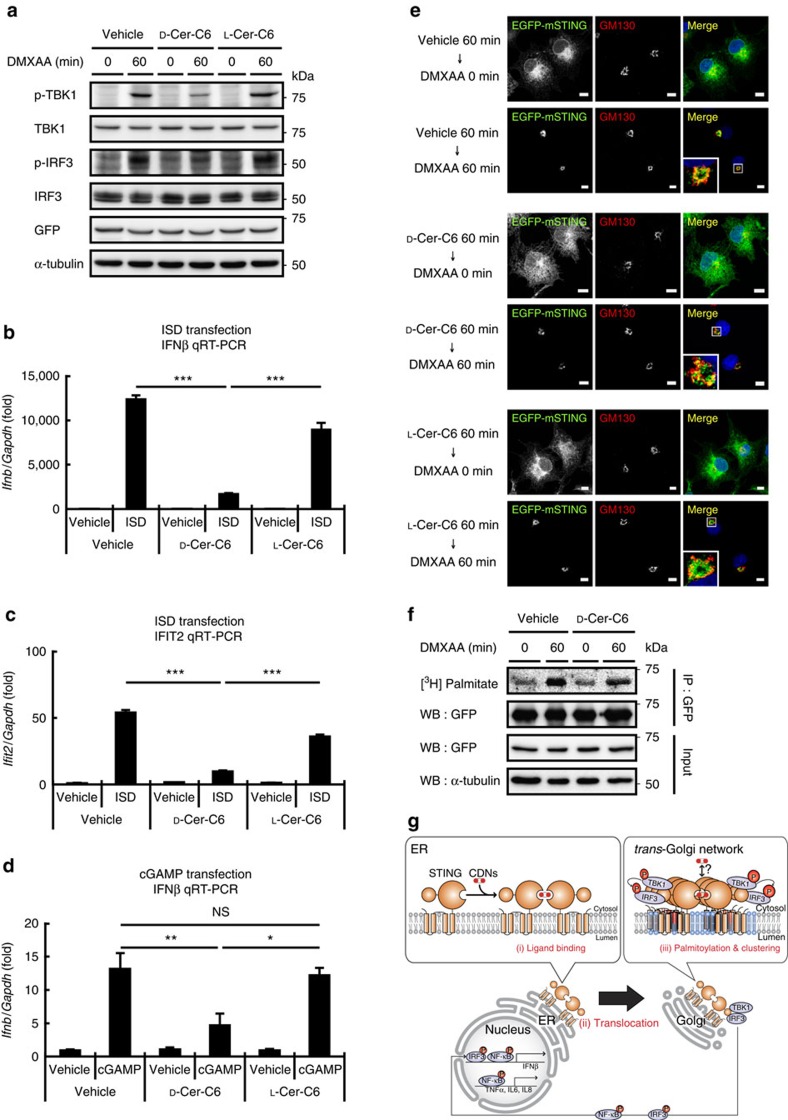
Disturbing Golgi lipid order suppresses the STING-dependent type I interferon response. (**a**) emsCOS-1 cells were treated with 20 μM D-ceramide-C6 (*N*-hexanoyl-D-erythro-sphingosine) or its non-metabolizable enantiomer L-ceramide-C6 (*N*-hexanoyl-L-erythro-sphingosine) for 1 h. Cells were stimulated with DMXAA for 1 h. Cell lysates were then prepared and analysed by western blot. (**b**–**d**) primary MEFs were treated with 20 μM D-ceramide-C6 or L-ceramide-C6 for 1 h. Cells were stimulated with ISD (**b**,**c**) or cGAMP (**d**) using Lipofectamine 2000 for 3 h. qRT-PCR of the expression of IFNβ (**b**,**d**) and Ifit2 (**c**) was performed. (**e**) emsCOS-1 cells processed as indicated were fixed, permeabilized and stained for GM130 (red). Nuclei were stained with DAPI (blue). Scale bars, 10 μm. (**f**) The effect of 20 μM D-ceramide-C6 on the palmitoylation of STING in emsCOS-1 cells. (**g**) a proposed mechanism of STING activation. (i) CDNs bind to STING at the ER, (ii) STING then translocates from the ER to the Golgi. (iii) STING is palmitoylated at the Golgi, and with the aid of the lipid rafts (light blue) present in the TGN, palmitoylated STING is clustered, which facilitates the STING signalling through the recruitment of TBK1 and IRF3. *P<0.01, **P<0.005, ***P<0.001, NS, not significant (one-way analysis of variance).

Protein palmitoylation has been implicated in the clustering of a number of proteins^30^ such as H-ras and Fas into lipid rafts (specific membrane domains enriched in cholesterol and sphingomyelin (SM)). Clustering of STING is proposed to bring TBK1 and IRF3 into close proximity, so that TBK1 can phosphorylate IRF3 (ref. 33). Palmitoylation of STING may facilitate the clustering of STING into lipid rafts at the TGN. Cholesterol is suggested to be enriched at the TGN, and cholesterol together with SM generated by SM synthase 1 are thought to form lipid rafts at the TGN^34^. Treatment of cells with D-ceramide-C6 disrupts lipid rafts at the Golgi by generating short-chain SM that disturbs the lipid order^34^. Of note, D-ceramide-C6 inhibited the STING-dependent phosphorylation of TBK1 and IRF3 (Fig. 5a) and the induction of *Ifnb* and *Ifit2* (Fig. 5b–d) without affecting the translocation of STING to the Golgi (Fig. 5e) or the palmitoylation of STING (Fig. 5f). D-ceramide-C6 had only marginal effects on the RLR pathway (Supplementary Fig. 14). L-ceramide-C6, a non-metabolizable enantiomer of D-ceramide-C6, did not inhibit the phosphorylation of TBK1 and IRF3. We propose that palmitoylation allows clustering of STING at the lipid rafts of the TGN, which facilitates the type I interferon response by bringing TBK1 and IRF3 closer to each other (Fig. 5g).

Proteins containing DHHC cysteine-rich domains (DHHC proteins) are protein palmitoyltransferases. The human genome encodes 23 DHHC proteins^35^. By overexpression of individual DHHC proteins in emsCOS-1 cells followed by metabolic labelling with [^3^H] palmitate, we found that DHHC3, DHHC7 and DHHC15 each increased the [^3^H] labelling of STING on stimulation (Supplementary Fig. 15a), and that this increase was dependent on their enzymatic activities (Supplementary Fig. 15b). This result may indicate that not all of the total STING was palmitoylated on stimulation, at least in COS-1 cells. DHHC3, DHHC7 and DHHC15 all localized to the Golgi (Supplementary Fig. 15c), suggesting that these DHHC proteins may contribute to the palmitoylation of C88/91 of STING at the Golgi. Since the mobility was not different between STING (WT) and STING (C88/91S) in SDS–PAGE, we could not determine how much of STING was palmitoylated.

STING, in addition to its association with SAVI, has also been associated with inflammations that are caused by dysregulated degradation of self-DNA^36^. Mice lacking DNase II or DNase III (Trex1) die during embryonic development or within 10 weeks of birth, respectively, with inflammation-related phenotypes. The lethality is completely rescued by the loss of STING^14^,^15^,^16^. Mutations of human DNase III are found in inflammatory disorders such as Aicardi–Goutieres syndrome and systemic lupus erythematosus^18^. Leakage of mitochondrial DNA into the cytoplasm, which occurs in many human diseases and ageing, induces STING-dependent inflammatory responses^37^. Our findings offer new opportunities to treat such cytosolic DNA-triggered inflammatory diseases by suppressing the palmitoylation of STING.

## Methods

### Antibodies

For the immunoprecipitation of endogenous STING, the rabbit polyclonal anti-STING antibody was used^23^ (dilution 1:100 for immunoprecipitation and dilution 1:50 for immunofluorescence). Other antibodies used in this study were as follows: mouse anti-GFP (JL-8, dilution 1:1,000; Clontech); mouse anti-GFP (3E6, dilution 1:500), Alexa 488-, 594- or 647-conjugated secondary antibodies (A21202, A21203, A21206, A21207, A31573, A11016, A21448, dilution 1:2,000; Life Technologies); rabbit anti-TBK1 (ab40676, dilution 1:1,000; Abcam); rabbit anti-phospho-TBK1 (D52C2, dilution 1:1,000 for western blotting, dilution 1:100 for immunofluorescence, dilution 1:50 for immunoelectron microscopy), rabbit anti-phospho-IRF3 (4D4G, dilution 1:1,000), rabbit anti-p65 (D14E12, dilution 1:1,000), and rabbit anti-phospho-p65 (93H1, dilution 1:1,000; Cell Signalling); rabbit anti-IRF3 (FL-425, dilution 1:200; Santa Cruz); rabbit anti-Rab11 (71-5300, dilution 1:100; Zymed); mouse anti-p62 (610382, dilution 1:1,000), mouse anti-calreticulin (612136, dilution 1:200) and mouse anti-GM130 (610823, dilution 1:1,000) (BD Biosciences); mouse anti-α-tubulin (DM1A, dilution 1:5,000; Sigma); sheep anti-mouse IgG antibody-HRP (NA9310V, dilution 1:4,000) and donkey anti-rabbit IgG antibody-HRP (NA9340V, dilution 1:4,000) (GE Healthcare); sheep anti-TGN46 (AHP500G, dilution 1:4,000 for immunofluorescence, dilution 1:200 for immunoelectron microscopy) and sheep anti-TGN38 (AHR499G, dilution 1:200) (Serotec); rabbit anti-STING antibody (19851-1-AP, dilution 1:1,000 for western blotting; Proteintech); rabbit anti-syntaxin5 (110,053, dilution 1:200; Synaptic Systems); mouse anti-HA (4B2, dilution 1:1,000 for western blotting and immunofluorescence; Wako); donkey anti-mouse IgG (H+L) conjugated with DyLight 405 (715-475-150, dilution 1:2,000), colloidal gold (CG) particle-conjugated donkey anti-rabbit antibody (12 nm; 711-205-152, dilution 1:20) and donkey anti-sheep antibody (6 nm; 713-195-147, dilution 1:20) (Jackson ImmunoResearch laboratories).

### Reagents

The following reagents were purchased from the manufacturers as noted: DMXAA, BFA, hydroxylamine-HCl, poly (I:C) and nocodazole (Sigma); 2-BP (Wako); Palmitate [9,10-3H(N)] (American Radiolabeled Chemicals Inc); 2′,3′-cGAMP (InvivoGen); D-ceramide-C6 (Cayman); L-ceramide-C6 (Matreya Inc). ISD (90-mer), used as dsDNA in this study, was prepared as follows^7^,^38^: equimolar amounts of oligonucleotides (sense: 5′- TACAGATCTACTAGTGATCTATGACTGATCTGTACATGATCTACATACAGATCTACTAGTGATCTATGACTGATCTGTACATGATCTACA -3′, antisense: 5′- TGTAGATCATGTACAGATCAGTCATAGATCACTAGTAGATCTGTATGTAGATCATGTACAGATCAGTCATAGATCACTAGTAGATCTGTA -3′) were annealed in PBS at 70 °C for 30 min before cooling to room temperature.

### Cell culture

COS-1 and HEK293T cells were purchased from the American Type Culture Collection (ATCC). Primary MEFs were obtained from embryos of WT or *Sting*^*−/−*^ mice at E13.5. BMDMs were differentiated from bone marrow cells using L929-conditioned medium^7^. COS-1, HEK293T and MEFs were cultured in DMEM supplemented with 10% foetal bovine serum/penicillin/streptomycin/glutamine in a 5% CO_2_ incubator. BMDMs were cultured in RPMI supplemented with 10% foetal bovine serum/penicillin/streptomycin/glutamine.

Reconstituted primary *Sting*^*−/−*^ MEFs with human STINGs were obtained using retrovirus^23^. Plat-E cells were transfected with pBabe-puro-STING and the medium that contains the retrovirus was collected. *Sting*^*−/−*^ MEFs were incubated with the medium and then selected with puromycin for a week.

emsCOS-1 cells were established using retrovirus transfection: HEK293T cells were transfected with pMXs-IP–EGFP–mSTING together with pCG-VSV-G and pCG-gag-pol, and the medium that contains the retrovirus was collected. COS-1 cells were incubated with the medium and then selected with puromycin for a week.

HEK293T cells that stably express human STINGs (WT, V147L or N154S) were established using retrovirus: Plat-E cells were transfected with pBabe-puro-STING together with pCG-VSV-G and the medium that contains the retrovirus was collected. HEK293T cells were incubated with the medium and then selected with puromycin for a week.

### PCR cloning

Mouse STING was amplified by PCR with complementary DNA (cDNA) derived from ICR mouse liver using the following primers: 5′- CCCGAATTCAATGCCATACTCCAACCTGCA -3′ (mSTING; sense primer, EcoRI site is underlined) and 5′- CCCGCGGCCGCTCAGATGAGGTCAGTGCGGA -3′ (mSTING; antisense primer, NotI site is underlined). The product encoding mSTING was introduced into pMXs-IP–GFP, to generate N-terminal GFP-tagged construct. STING Cys mutants were generated by site-directed mutagenesis.

### Luciferase assay

HEK293T cells seeded on 24-well plates were transiently transfected with luciferase reporter plasmid (100 ng), pRL-TK (10 ng) as internal control and STING-expression plasmid in pBabe vector (200 ng). Twenty four hours after the transfection, the luciferase activity in the total cell lysate was measured.

cGAMP stimulation of STING was performed as follows^39^: Cell medium was aspirated and replaced with 200 μl of 300 ng ml^−1^ cGAMP, 50 mM HEPES (pH 7.0), 100 mM KCl, 3 mM MgCl_2_, 0.1 mM DTT, 85 mM sucrose, 0.2% BSA, 1 mM ATP and 10 μg ml^−1^ digitonin. Cells were incubated for 30 min at 37 °C. The medium was then replaced with 500 μl of fresh growth media without antibiotics. Eight hours after cGAMP stimulation, luciferase activity in the total cell lysate was measured.

### qRT-PCR

Total RNA was extracted from cells using Isogen II (Nippongene), purified using High Pure RNA Tissue kit (Roche) and reverse-transcribed using the High Capacity cDNA Reverse Transcription kit (Applied Biosystems). Quantitative real-time PCR (qRT-PCR) was performed using LightCycler 480 SYBR Green I Master (Roche) and LightCycler 480 (Roche). The sequences of the primers were as follows. 5′- GACCAACAAGTGTCTCCTCCAAA -3′ (human *IFNB*; sense primer) and 5′- AGCAAGTTGTAGCTCATGGAAAGAG -3′ (human *IFNB*; antisense primer); 5′- AGGTGCAGTTTTGCCAAGGA -3′ (human *IL8*; sense primer) and 5′- TTTCTGTGTTGGCGCAGTGT -3′ (human *IL8*; antisense primer); 5′- ACTTTGGAGTGATCGGCCCCCAGA -3′ (human *TNF*; sense primer) and 5′- GCTTGTCACTCGGGGTTCGAGAAGA -3′ (human *TNF*; antisense primer); 5′- GCCAAGGTCATCCATGACAACT -3′ (human *GAPDH*; sense primer) and 5′- GAGGGGCCATCCACAGTCTT -3′ (human *GAPDH*; antisense primer); 5′- CAGCTCCAAGAAAGGACGAAC -3′ (mouse *Ifnb*; sense primer) and 5′- GGCAGTGTAACTCTTCTGCAT -3′ (mouse *Ifnb*; antisense primer); 5′- TAGTCCTTCCTACCCCAATTTC -3′ (mouse *Il6*; sense primer) and 5′- TTGGTCCTTAGCCACTCCTTC -3′ (mouse *Il6*; antisense primer); 5′- AGGTCGGTGTGAACGGATTTG -3′ (mouse *Gapdh*; sense primer) and 5′- TGTAGACCATGTAGTTGAGGTCA -3′ (mouse *Gapdh*; antisense primer). Target gene expression was normalized on the basis of GAPDH content.

### Immunocytochemistry

Cells were fixed with 4% paraformaldehyde (PFA) in PBS at room temperature for 15 min, permeabilized with 0.1% Triton X-100 in PBS at room temperature for 5 min and quenched with 50 mM NH_4_Cl in PBS at room temperature for 10 min. After blocking with 3% BSA in PBS, cells were incubated with primary antibodies, then with secondary antibodies conjugated with Alexa fluorophore.

### Confocal microscopy

Confocal microscopy was performed using a TCS SP8 (Leica) with a 63 × 1.2 Plan-Apochromat water immersion lens.

### Nocodazole treatment

Cells were treated with nocodazole (20 μM) for 30 min, stimulated with DMXAA (25 μg ml^−1^) in the presence of nocodazole for 100 min, then fixed and stained.

### Immunoelectron microscopy

emsCOS-1 cells were stimulated with DMXAA for 1 h and fixed with 4% PFA (1.04005.1000, MERCK), 4% sucrose and 0.1 M phosphate buffer (pH 7.2) for 10 min at room temperature and then 30 min at 4 °C. After rinsing with 7.5% sucrose and 0.1 M phosphate buffer (pH 7.4), the cells were scraped and embedded in 10% gelatin (G2500, Sigma) and 0.1 M phosphate buffer (pH 7.4). The cell blocks were cut into small pieces (about 1 mm cube), which were infused overnight with 20% polyvinylpyrrolidone (PVP10, Sigma-Aldrich), 1.84 M sucrose, 10 mM Na_2_CO_3_ and 0.08 M phosphate buffer (pH 7.4) followed by rapid freezing in liquid nitrogen^40^. Ultrathin cryosections were prepared using an ultramicrotome (EM UC7, Leica) equipped with a cryochamber (EM FC7, Leica). They were incubated with 1% BSA and PBS for 20 min at room temperature, and then with two primary antibodies, anti-p-TBK1 (1:50, D52C2) and anti-TGN46 (1:200, AHP500GT, Bio-Rad), for 3 days at 4 °C. After incubation with two CG-conjugated secondary antibodies, 6 nm CG-donkey anti-sheep (1:20, 713-195-147) and 12 nm CG-donkey anti-rabbit (1:20, 711-205-152, Jackson ImmunoResearch), for 1 h at room temperature, they were fixed with 2% glutaraldehyde (G017/1, TAAB) and PBS for 5 min. They were stained with 2% uranyl acetate for 5 min and embedded in 0.17% uranyl acetate and 0.33% polyvinyl alcohol (P8136, Sigma-Aldrich). After drying up, sections were observed using an electron microscope (JEM1200EX, JEOL).

### Western blotting

Proteins were separated in polyacrylamide gel and then transferred to polyvinylidene difluoride membranes (Millipore). These membranes were incubated with primary antibodies, followed by secondary antibodies conjugated to peroxidase. The proteins were visualized by enhanced chemiluminescence using a LAS-4000 (GE Healthcare). Uncropped images are shown in Supplementary Fig. 16.

### Metabolic labelling with [^3^H] palmitate

emsCOS-1 cells were incubated in DMEM containing 0.1% essentially fatty acid-free BSA for 1 h at 37 °C, and then metabolically labelled with 0.1 mCi ml^−1^ [^3^H] palmitate at 37 °C. One hour after the labelling, DMXAA (final 25 μg ml^−1^) was added to the cell medium. After incubation for appropriate times, cells were washed with ice-cold PBS, scraped in immunoprecipitation buffer composed of 50 mM HEPES-NaOH (pH 7.2), 150 mM NaCl, 5 mM EDTA, 1% SDS, 1% Triton X-100, protease inhibitors (1 mM PMSF, 10 μg ml^−1^ leupeptin, 10 μg ml^−1^ pepstatin, 10 μg ml^−1^ aprotinin) and phosphatase inhibitors (8 mM NaF, 12 mM beta-glycerophosphate, 1 mM Na_3_VO_4_, 1.2 mM Na_2_MoO_4_, 5 μM cantharidin and 2 mM imidazole). The lysates were then sonicated on ice and diluted to 0.1% SDS. After centrifugation at 15,000 r.p.m. for 10 min at 4 °C, the resultant supernatants were incubated for overnight at 4 °C with anti-GFP (3E6), and then incubated for 3 h with protein G Sepharose fast flow (GE Healthcare). The beads were washed four times with immunoprecipitation wash buffer (50 mM HEPES-NaOH (pH 7.2), 150 mM NaCl, 0.1% Triton X-100) and eluted with 2 × Laemmli sample Buffer. The immunoprecipitated proteins were separated with SDS–PAGE and transferred to PVDF membrane, then autoradiographed with BAS-IP TR2040 and Typhoon9000 (GE Healthcare). In the case of MEF cells, immunoprecipitation buffer without SDS and anti-STING polyclonal antibody were used. For hydroxylamine treatment^32^, PVDF membranes were soaked in 1 M hydroxylamine (pH 7.0)/1% SDS for 3 h at room temperature.

### Virus infection

HSV-1 (KOS strain) was purchased from ATCC. HSV-1 g34.5 was kindly provided by Dr Bernard Roizman (The University of Chicago).

### Statistical analyses

Error bars displayed throughout this study represent s.e.m. unless otherwise indicated, and were calculated from triplicate or quadruplicate samples. Statistical significance was determined with one-way analysis of variance followed by Tukey–Kramer *post hoc* test.; *P<0.05; **P<0.01; ***P<0.001; NS, not significant (*P*>0.05). Data shown are representative of two to three independent experiments, including microscopy images and western blots.

### Data availability

The data that support the findings of this study are available from the corresponding author upon request.

## Additional information

**How to cite this article**: Mukai, K. *et al*. Activation of STING requires palmitoylation at the Golgi. *Nat. Commun.* 7:11932 doi: 10.1038/ncomms11932 (2016).

## Supplementary Material

Supplementary Information Supplementary figures 1-16
