# Supplementary material for: Activation of STING requires palmitoylation at the Golgi
Source: Nat Commun. 2016 Jun 21;7:11932. doi: 10.1038/ncomms11932 (PMC4919521; doi:10.1038/ncomms11932)
Supplement: Supplementary Information — Supplementary figures 1-16 [file ncomms11932-s1.pdf]

Supplementary Figure 1

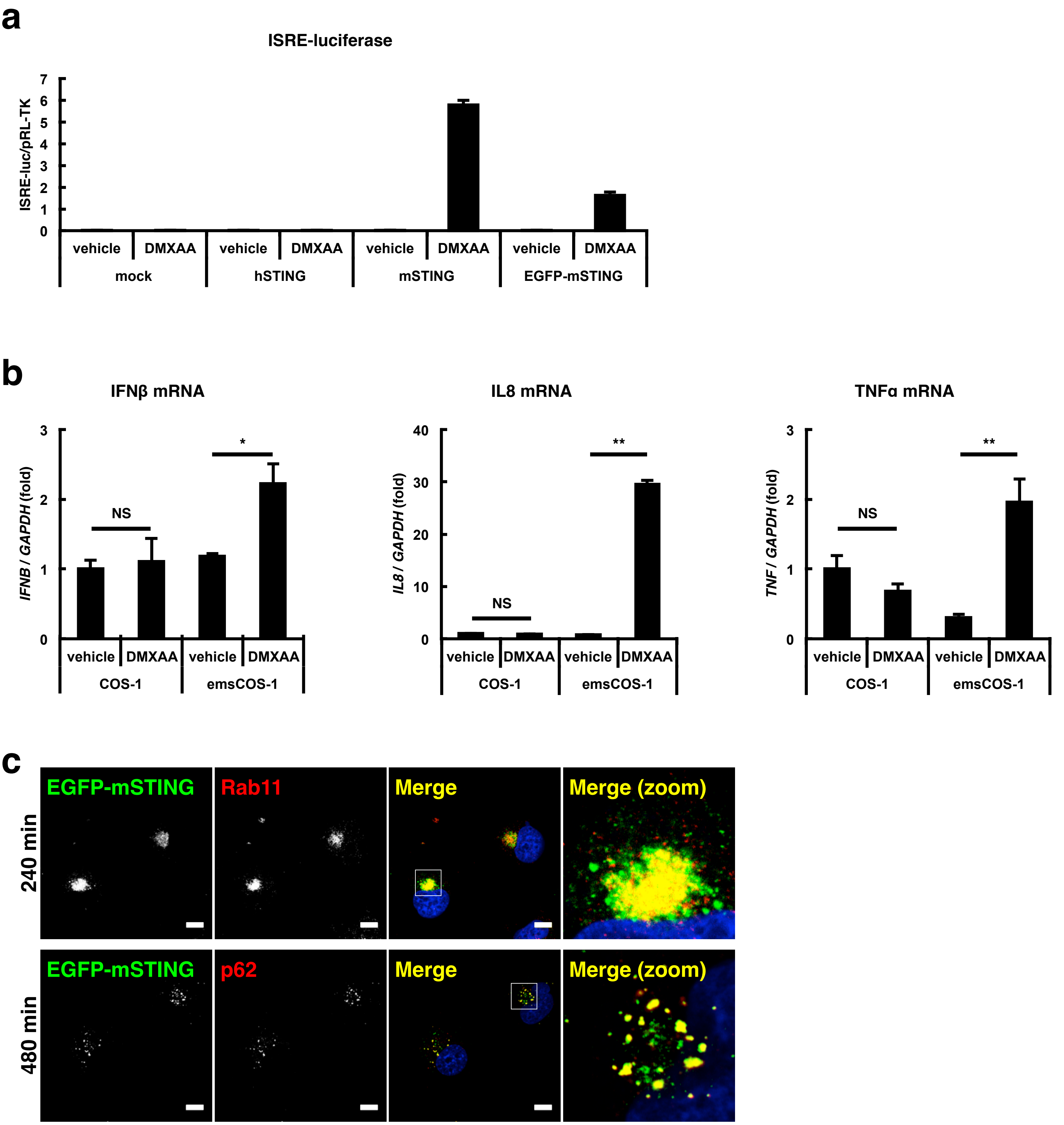

Supplementary Figure 1. DMXAA activates the STING pathway in emsCOS-1 cells that stably express EGFP-mouse STING (emsCOS-1). a, HEK293T cells were transfected as indicated, together with an ISRE (also known as PRDIII or IRF-E)-luciferase reporter, followed by stimulation of DMXAA for 12 h. Luciferase activity was then measured. Data represent mean ± s.e.m. of two independent experiments. **b**, qRT–PCR of the expression of indicated genes in COS-1 or emsCOS-1 cells that were stimulated with DMXAA for 12 h (IFNβ) or 6 h (IL8 and TNFα). Data represent mean ± s.e.m. from three independent experiments. *P < 0.01, **P < 0.005, NS, not significant (one-way ANOVA). **c**, emsCOS-1 cells were stimulated with DMXAA for the indicated times, fixed, permeabilized, and stained for Rab11 (a recycling endosomal protein) or p62 (an autophagy-associated protein). Nuclei were stained with DAPI (blue). Scale bars, 10 μm.

Supplementary Figure 2

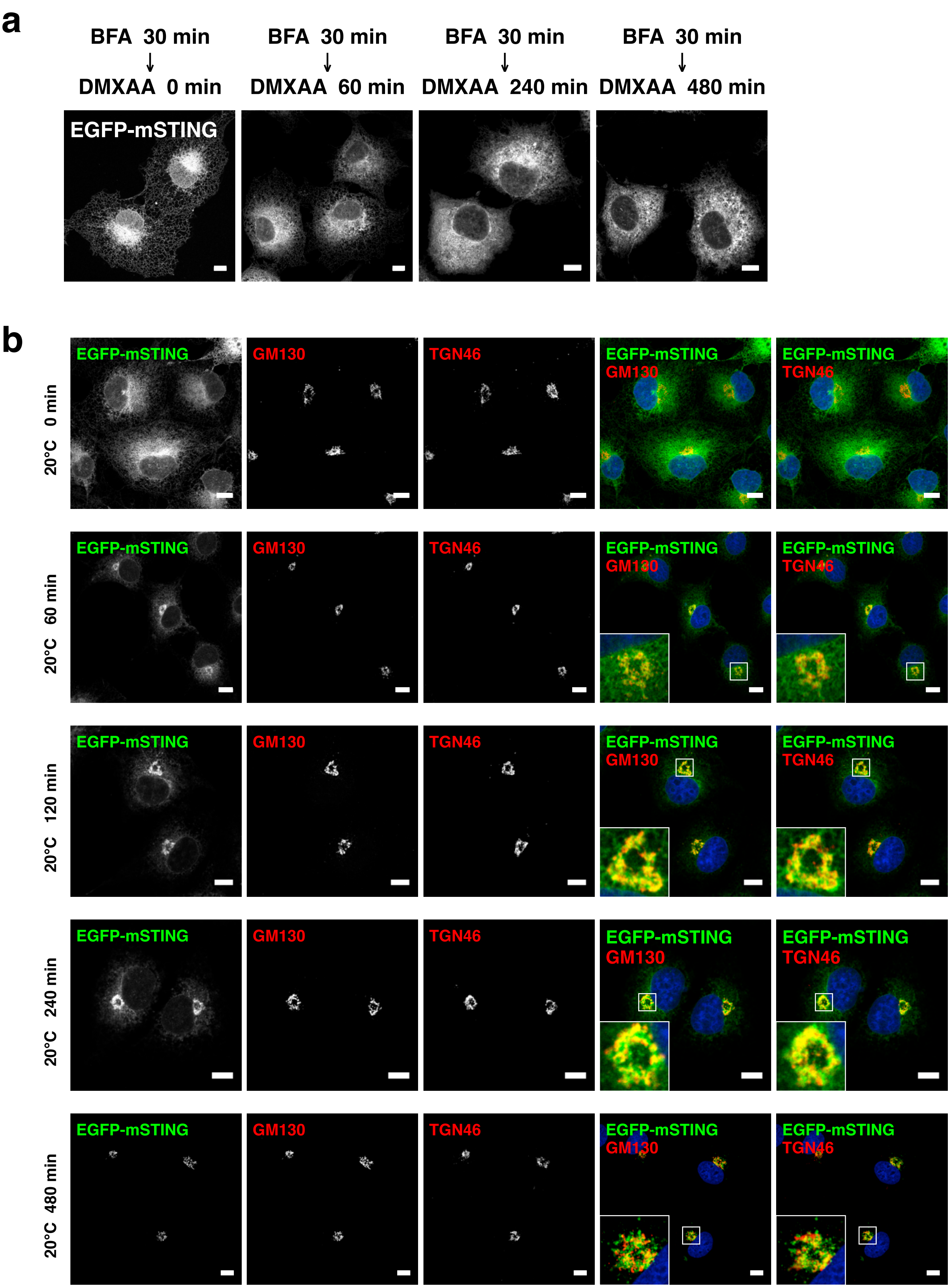

Supplementary Figure 2. Effects of BFA and 20 °C treatment on STING traffic. a, emsCOS-1 cells were treated with BFA (0.3 μg mL^{-1}) for 30 min. Cells were stimulated with DMXAA for the indicated times and then fixed. **b**, emsCOS-1 cells were precultured at 20 °C for 30 min. Cells were then stimulated with DMXAA for the indicated times, fixed, and stained for GM130 (a CGN protein) or TGN46 (a TGN protein). Scale bars, 10 μm.

Supplementary Figure 3

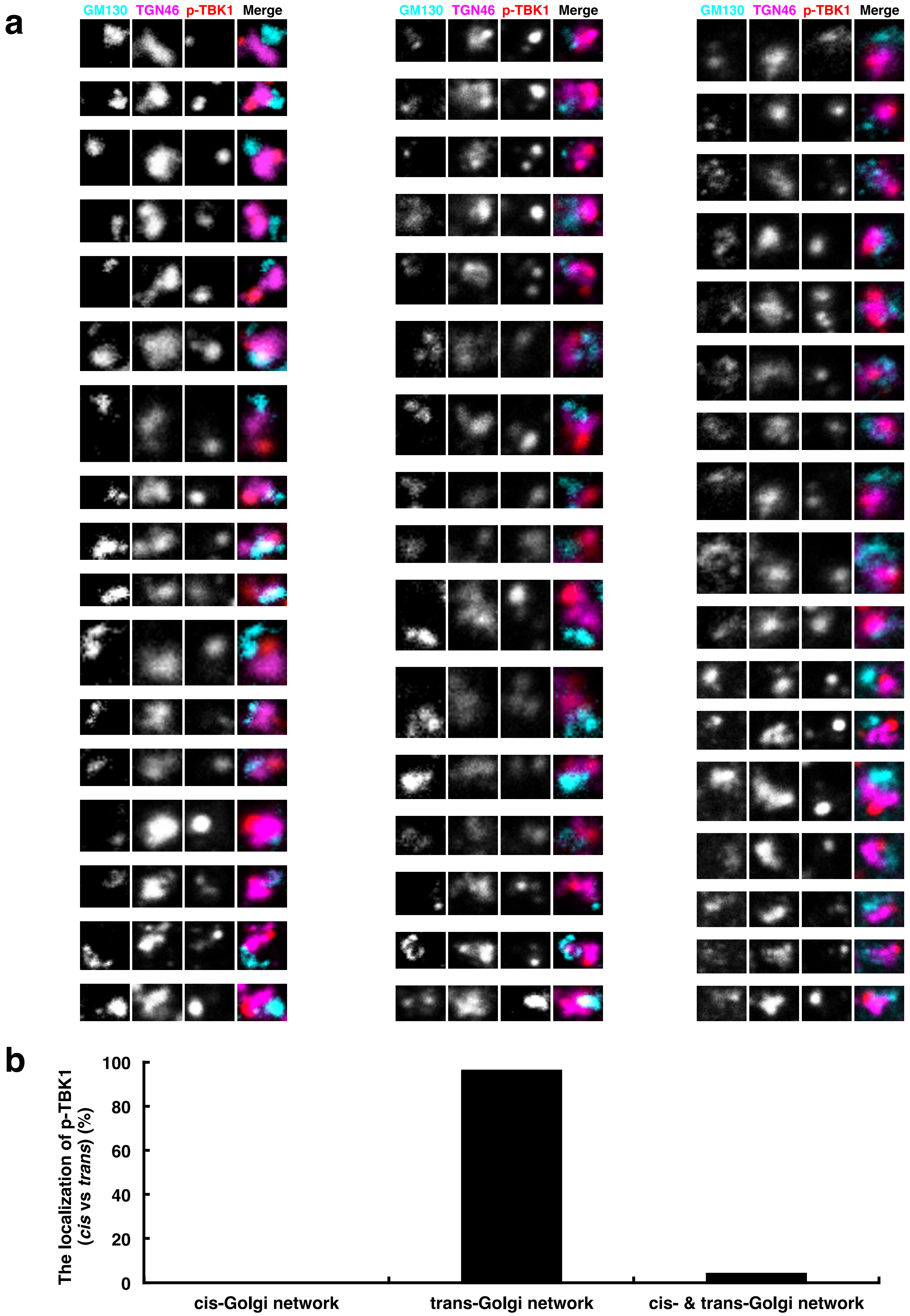

Supplementary Figure 3. Quantification of p-TBK1 localization in the Golgi, related to Figure 1e. a, 50 mini-Golgi stacks in emsCOS-1 cells treated with nocodazole and DMXAA (8 cells). Cells were triple labelled with phosphorylated TBK1 (red), GM130 (a CGN protein, cyan) and TGN46 (a TGN protein, magenta). **b**, Quantification of p-TBK1 localization in the mini-Golgi in (**a**).

Supplementary Figure 4

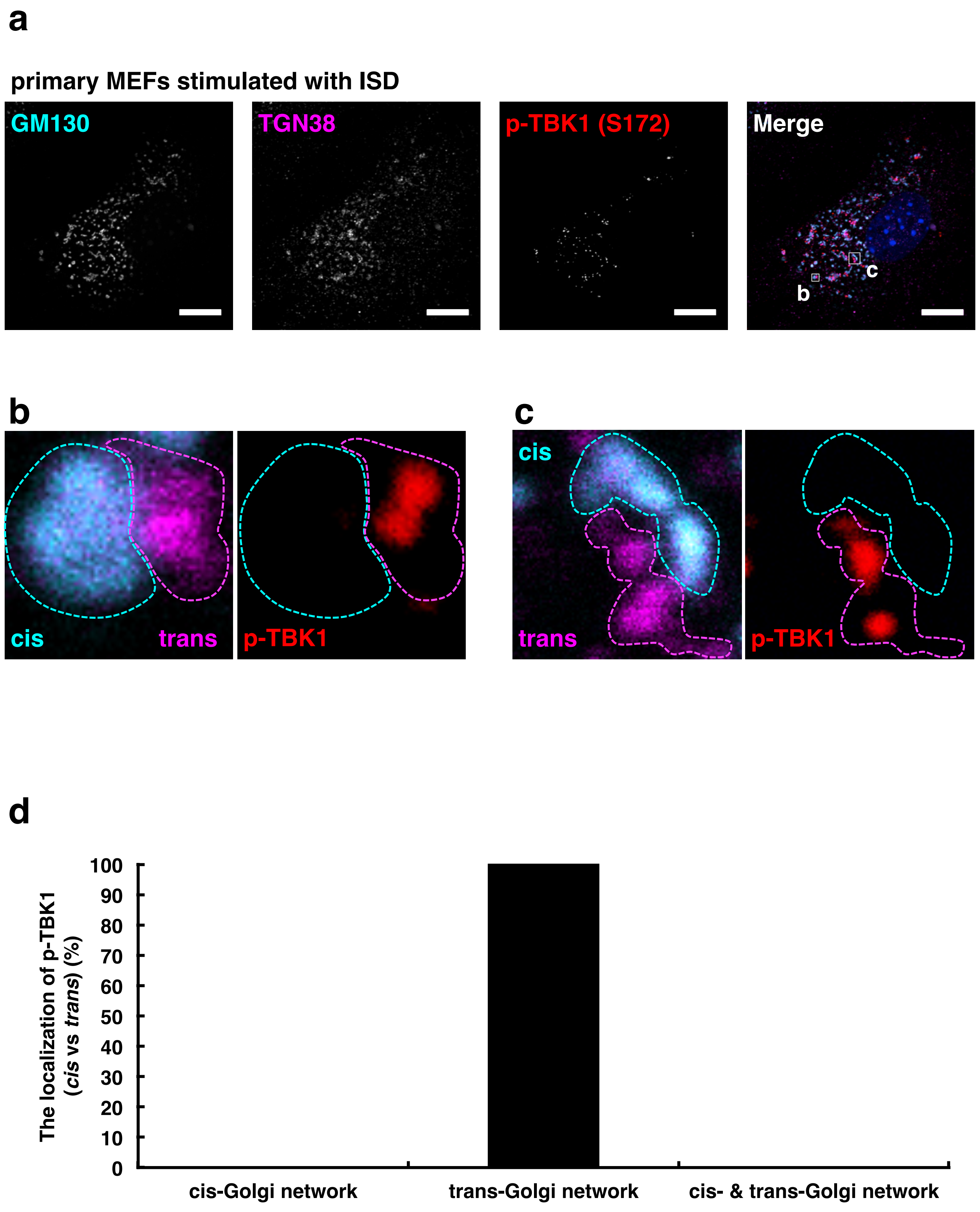

Supplementary Figure 4. Phosphorylated TBK1 is localized to the TGN in ISD-stimulated primary MEFs. a, Immunostaining of phosphorylated TBK1 (red) in cells treated with nocodazole and ISD. GM130 (a CGN protein, cyan) and TGN46 (a TGN protein, magenta) were co-stained. Scale bars, 10 μm**. b-c**, The mini-Golgi stacks indicated by the boxes in (**a**) were magnified. The *cis* and *trans*-regions of the mini-Golgi stacks were outlined with dashed line. **d**, Quantification of p-TBK1 localization in 27 mini-Golgi stacks.

Supplementary Figure 5

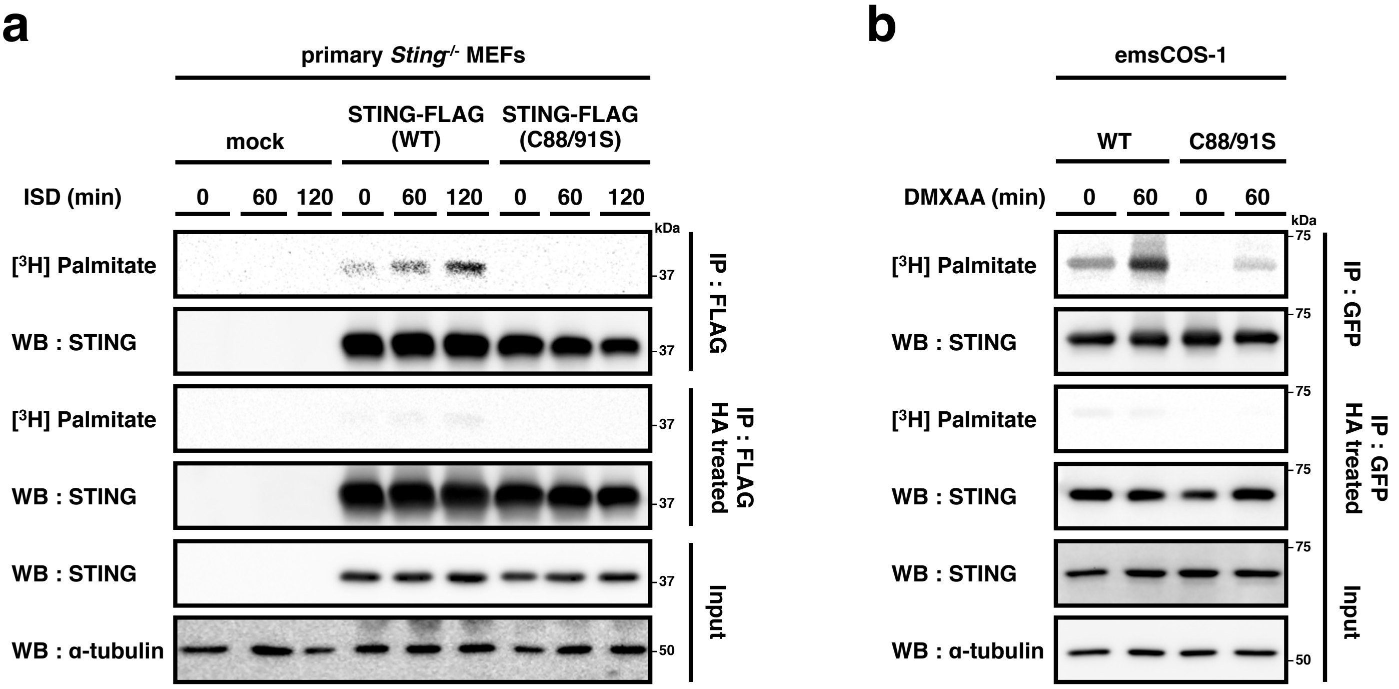

Supplementary Figure 5. Hydroxylamine treatment of STING labelled with [^{3}H]-palmitate. a, Primary *Sting*$^{-/-}$ MEFs were reconstituted with human STING-FLAG (WT or C88/91S) using retroviruses. The cells were and metabolically labelled, stimulated with ISD for the indicated times, and analyzed by Western blot and autoradiography. For acyl thioester analysis, membranes were soaked in 1 M hydroxylamine. **b**, COS-1 cells that stably express EGFP-mouse STING (WT or C88/91S) were stimulated with DMXAA, and analyzed as in (**a**).

Supplementary Figure 6

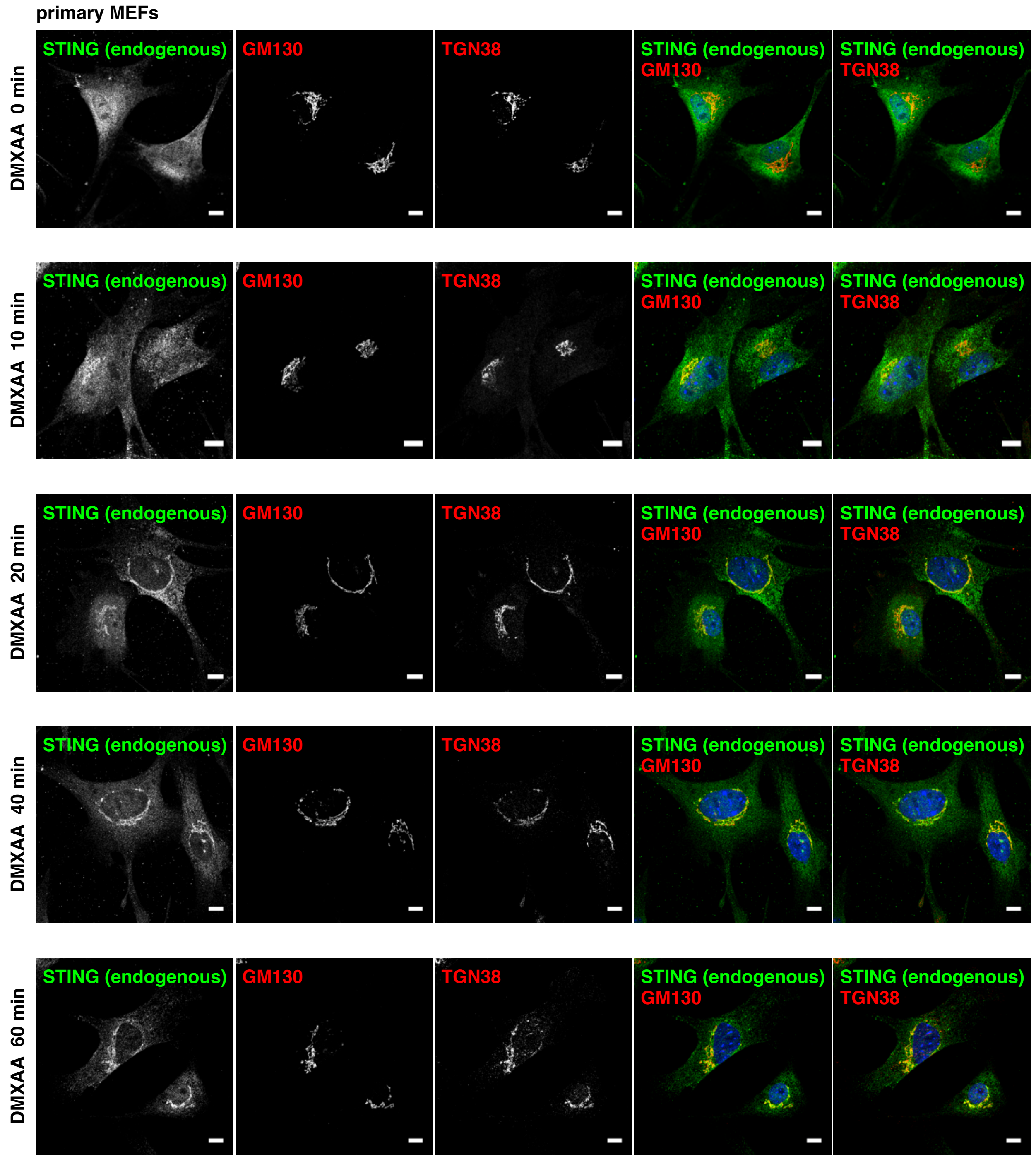

Supplementary Figure 6. Translocation of endogenous STING in primary MEFs. Primary MEFs were stimulated with DMXAA for the indicated times, fixed, permeabilized, and stained for STING, GM130, and TGN38 as indicated. Nuclei were stained with DAPI (blue). Scale bars, 10 µm.

Supplementary Figure 7

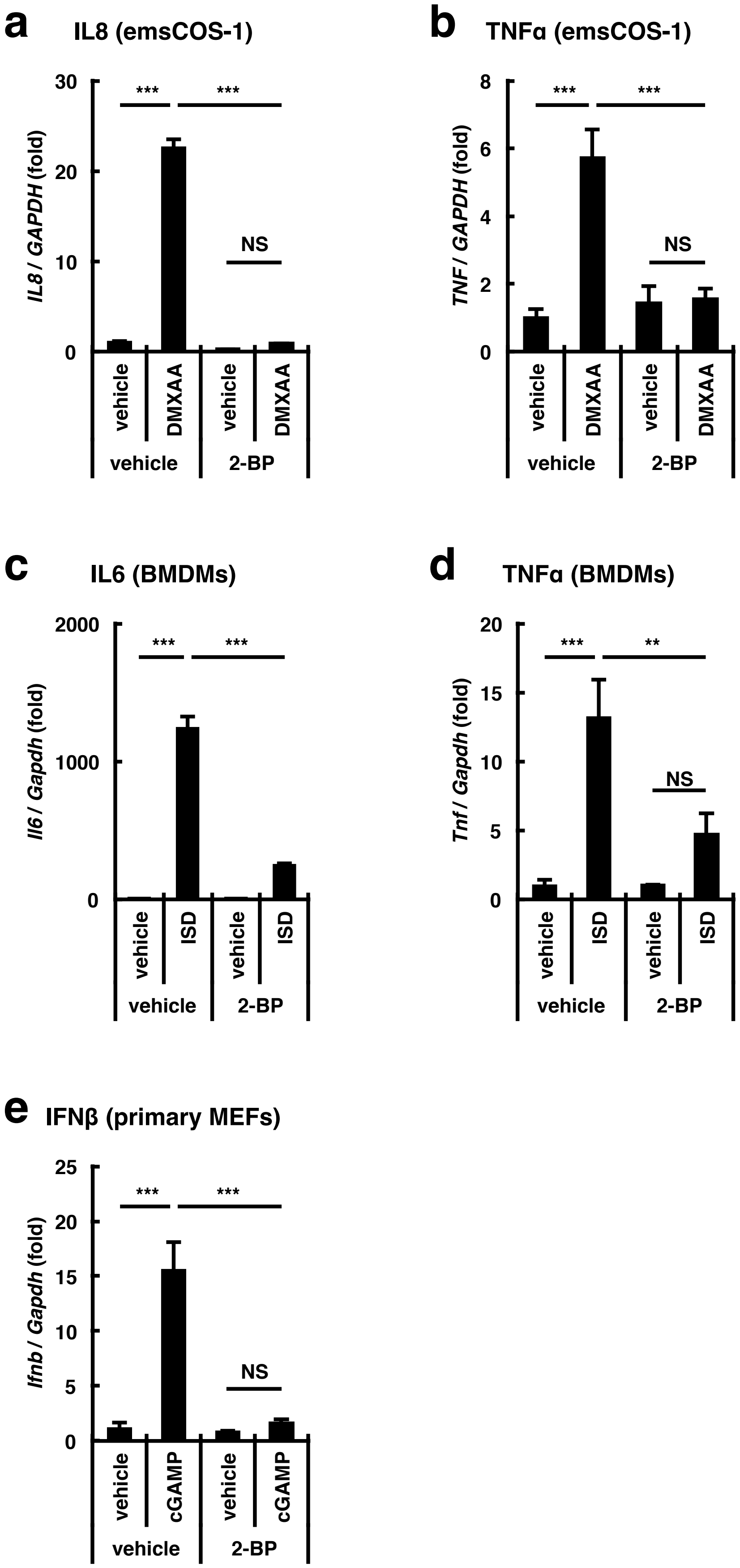

Supplementary Figure 7. 2-BP supresses the STING-dependent type I interferon response and inflammatory response, related to Figure 2(d-f). a and **b**, qRT–PCR of the expression of IL8 and TNFα in emsCOS-1 cells that were pretreated with vehicle or 50 μM 2-BP for 1 h and then stimulated with DMXAA for 6 h. **c** and **d**, qRT–PCR of the expression of IL6 and TNFα in BMDMs that were pretreated with vehicle or 50 μM 2-BP for 1 h and then stimulated with ISD for 3 h. **e**, qRT–PCR of the expression of IFNβ in primary MEFs that were pretreated with vehicle or 50 μM 2-BP for 1 h and then stimulated with cGAMP for 3 h. $^{**}P < 0.005$, $^{***}P < 0.001$, NS, not significant (one-way ANOVA).

Supplementary Figure 8

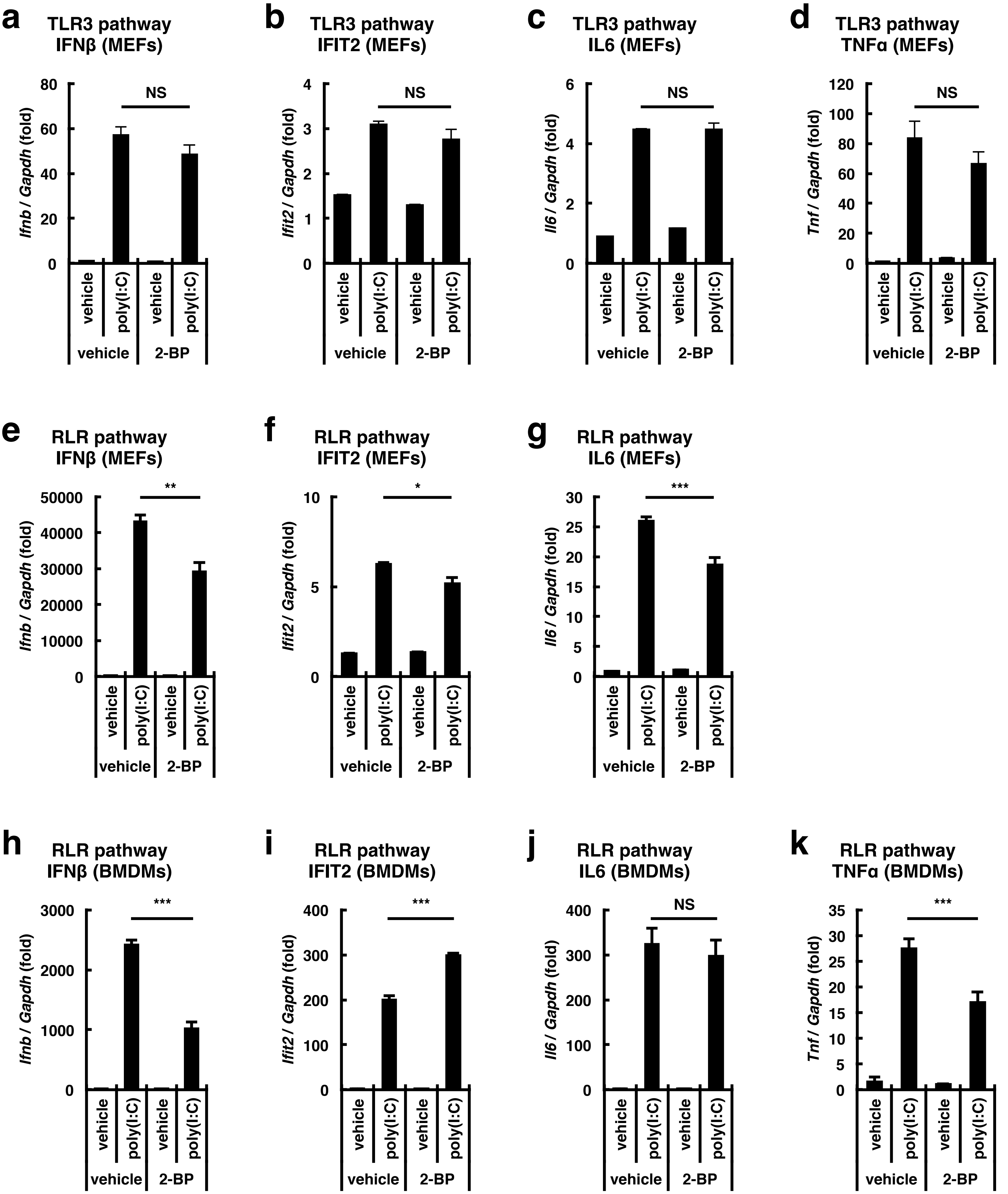

Supplementary Figure 8. The effect of 2-BP on the TLR3 and RLR pathways. a-d, qRT–PCR of the expression of indicated genes in MEFs that were pretreated with vehicle or 50 μM 2-BP for 1 h and then stimulated with the supplementation of poly (I:C) for 3 h to activate the TLR3 pathway. **e-g**, qRT–PCR of the expression of indicated genes in MEFs that were pretreated with vehicle or 50 μM 2-BP for 1 h and then stimulated with the transfection of poly (I:C) for 3 h to activate the RLR pathway. **h-k**, qRT–PCR of the expression of indicated genes in BMDMs that were pretreated with vehicle or 50 μM 2-BP for 1 h and then stimulated with the transfection of poly (I:C) for 3 h to activate the RLR pathway. *P < 0.01, **P < 0.005, ***P < 0.001, NS, not significant (one-way ANOVA).

Supplementary Figure 9

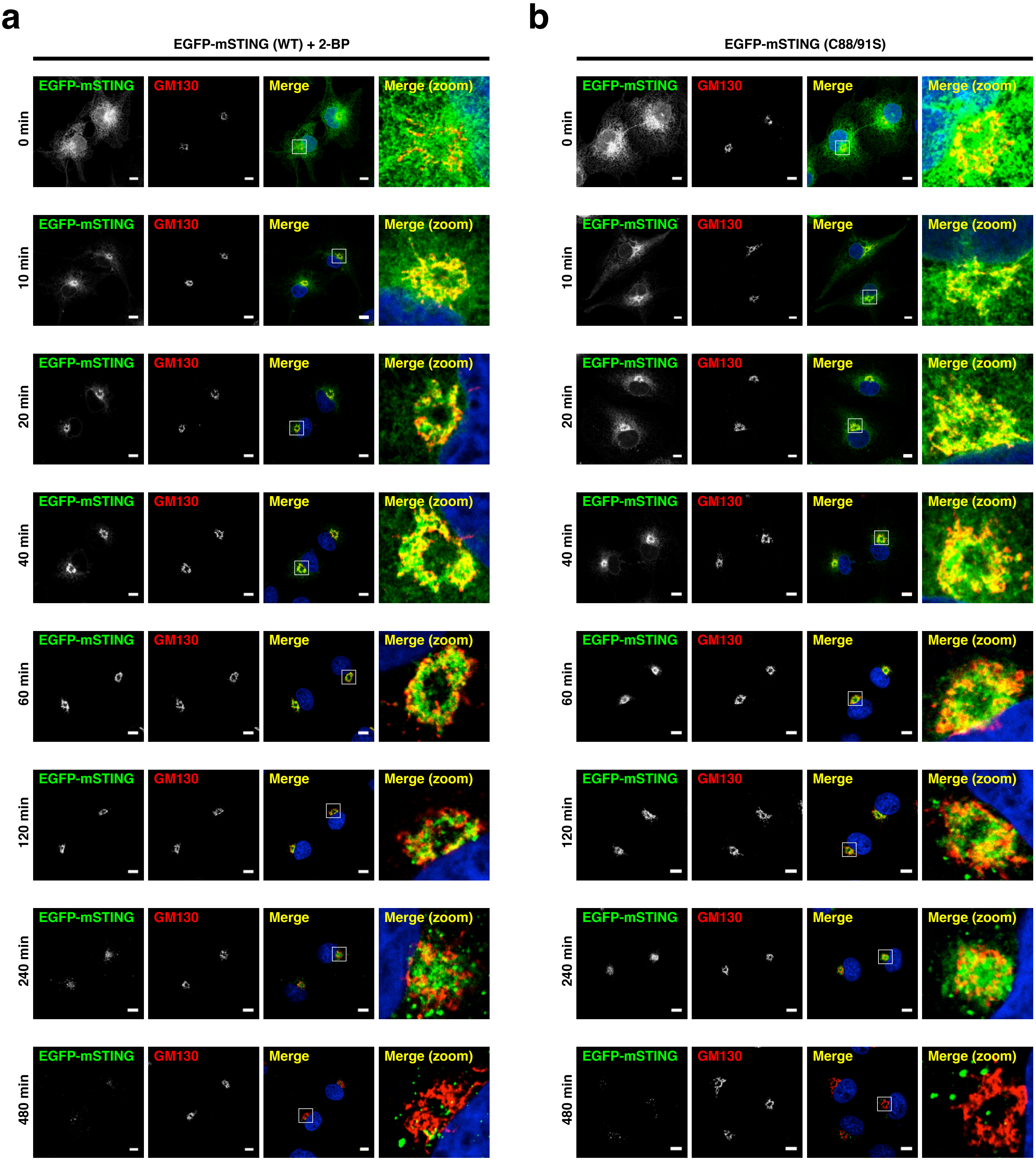

Supplementary Figure 9. Palmitoylation of STING is not essential for the post-ER trafficking. a, emsCOS-1 cells were treated with 50 μM 2-BP for 60 min, stimulated with DMXAA for the indicated times, fixed, and stained for GM130. **b**, COS-1 cells that stably express EGFP-mSTING (C88/91S) were stimulated with DMXAA for the indicated times, fixed, and stained for GM130. Scale bar, 10 μm.

Supplementary Figure 10

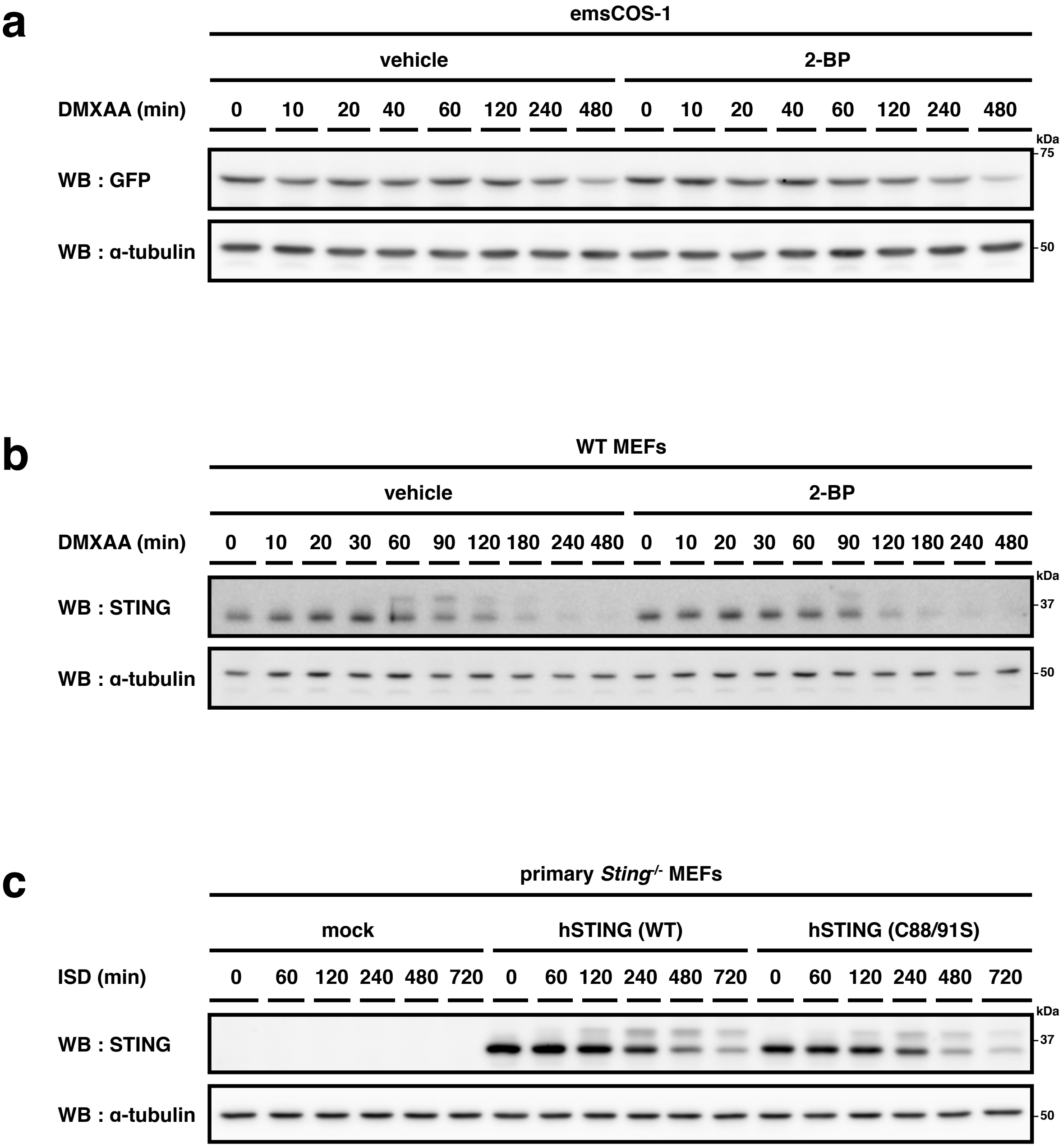

Supplementary Figure 10. Treatment with 2-BP or the mutation of C88/91 did not affect the degradation rate of STING. a, Western blots of cell lysates of emsCOS-1 cells that were pretreated with vehicle or 50 μM 2-BP for 1 h and then stimulated with DMXAA for the indicated times. **b**, Western blots of cell lysates of MEFs that were pretreated with vehicle or 50 μM 2-BP for 1 h and then stimulated with DMXAA for the indicated times. **c**, Primary *Sting*$^{-/-}$ MEFs were reconstituted with hSTING (WT) or hSTING (C88/91S). Cells were stimulated with ISD for the indicated times and the cell lysates were analyzed by Western blots.

Supplementary Figure 11

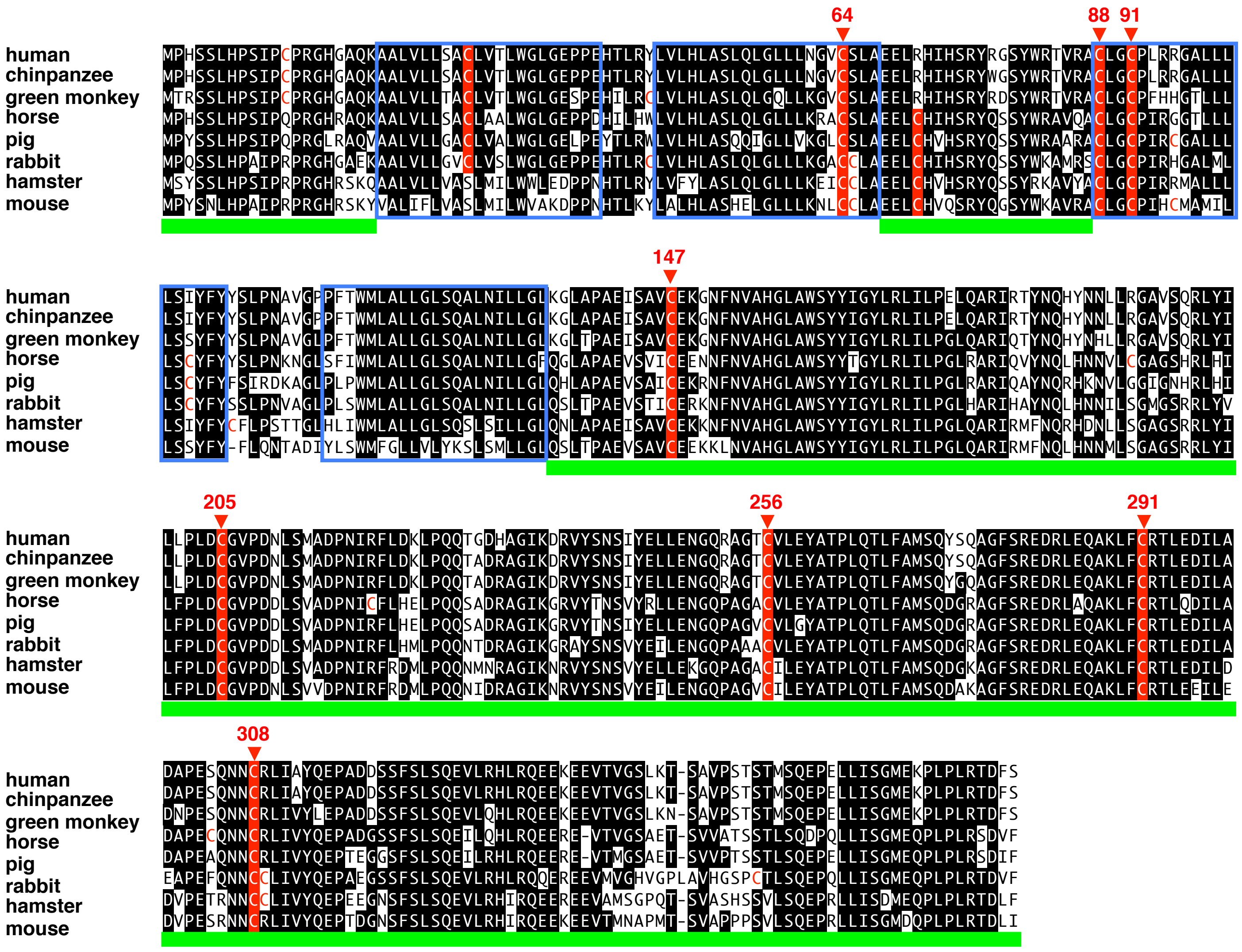

Supplementary Figure 11. Conserved cysteine residues of STING in mammals. Alignment of the amino acid sequence of mammalian STING proteins. Red letter indicates cysteine residue, blue box indicates transmembrane region, and green underline indicates the cytosolic region.

Supplementary Figure 12

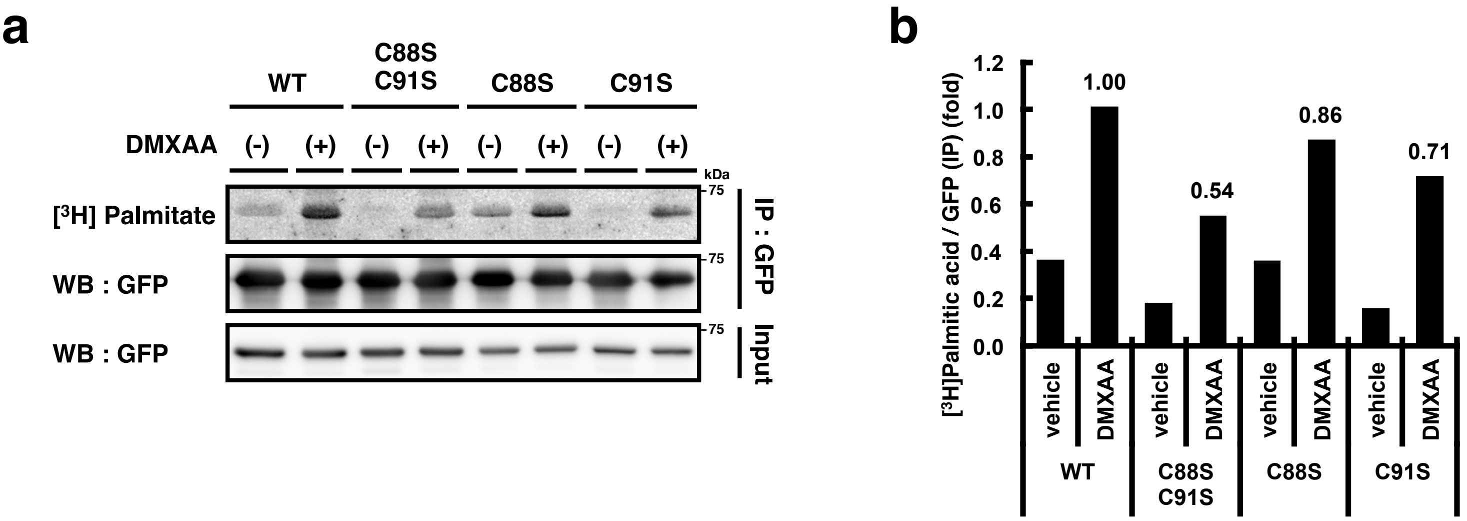

Supplementary Figure 12. Effects of single mutation of Cys88 or Cys91 on STING palmitoylation. a, COS-1 cells that stably express EGFP-mouse STING with the indicated Cys substitutions were metabolically labelled, and analyzed by Western blot and autoradiography. **b**, The band intensities (**a**) were quantified and [^{3}H palmitate]/[GFP] were calculated. The data were normalized to the value of WT-STING with DMXAA treatment.

Supplementary Figure 13

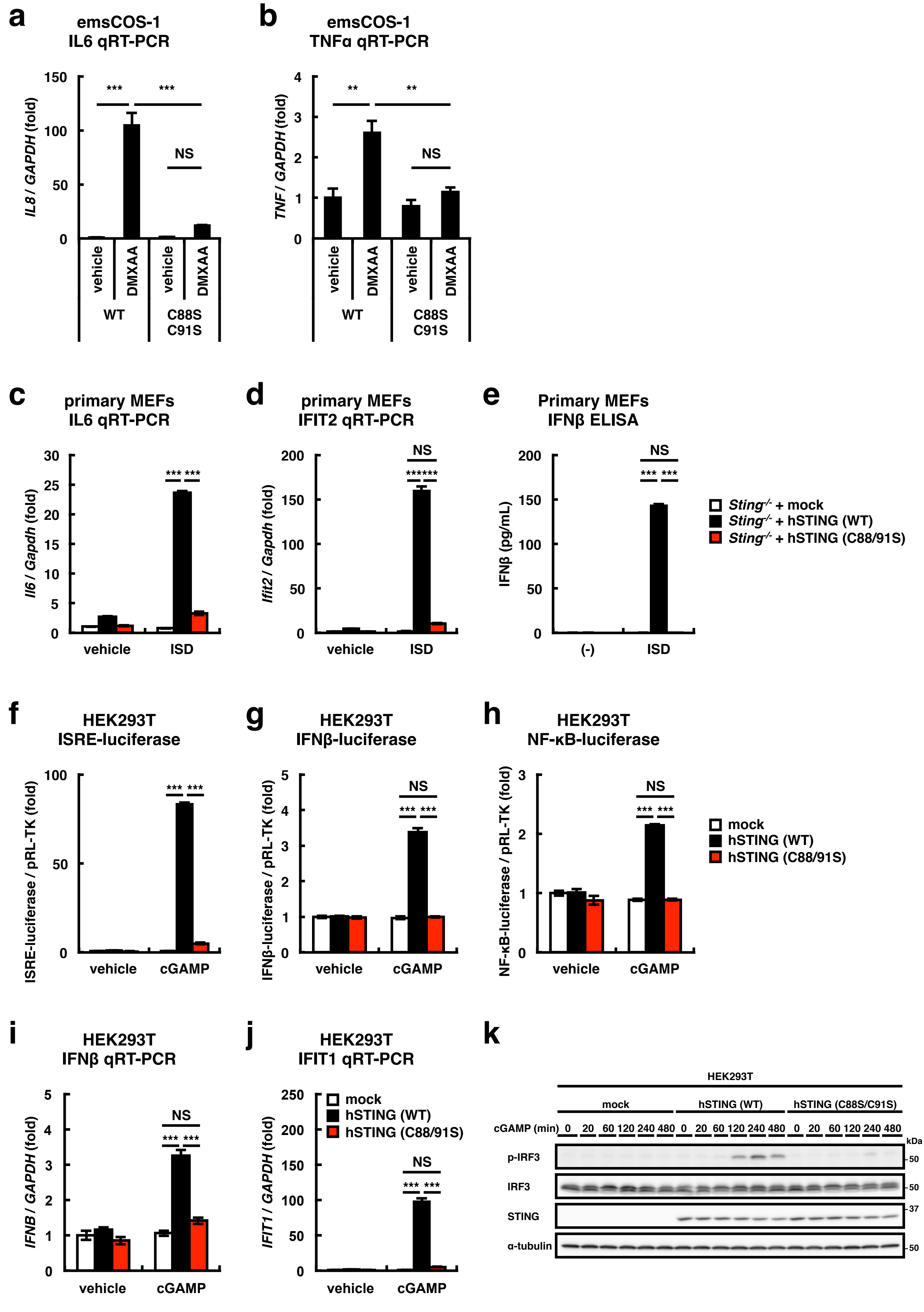

Supplementary Figure 13. STING C88/91S mutant is unable to induce the type I interferon response elicited by DMXAA, cGAMP, or ISD. a and **b**, COS-1 cells that stably express STING (WT or C88/91S) were stimulated with DMXAA for 6 h. qRT–PCR of the expression of the indicated genes was then performed. **c**-**e**, Primary *Sting*$^{-/-}$ MEFs were reconstituted with human STING variants using retroviruses, and stimulated with ISD for 6 h (**c** and **d**) or for 12 h (**e**). In (**c** and **d**), qRT-PCR of the expression of the indicated genes was performed. In (**e**), the concentration of IFNβ was measured by ELISA. **f**-**h**, HEK293T cells were transfected as indicated, together with an ISRE (also known as PRDIII or IRF-E)-luciferase reporter (**f**), IFNβ-luciferase reporter (**g**), and NF-κB-luciferase reporter (**h**), followed by stimulation of cGAMP for 8 h. Luciferase activity was then measured. **i** and **j**, HEK293T cells were transfected as indicated, followed by stimulation of cGAMP for 8 h. qRT–PCR of the expression of the indicated genes was then performed. **k**, HEK293T cells were transfected as indicated. Cell lysates were prepared the indicated times after cGAMP stimulation, and analyzed by Western blot. Data represent mean ± s.e.m. from four (**f**-**h**) or three (**a-e**, **i**, and **j**) independent experiments. $^{**}P < 0.005$, $^{***}P < 0.001$, NS, not significant (one-way ANOVA).

Supplementary Figure 14

a

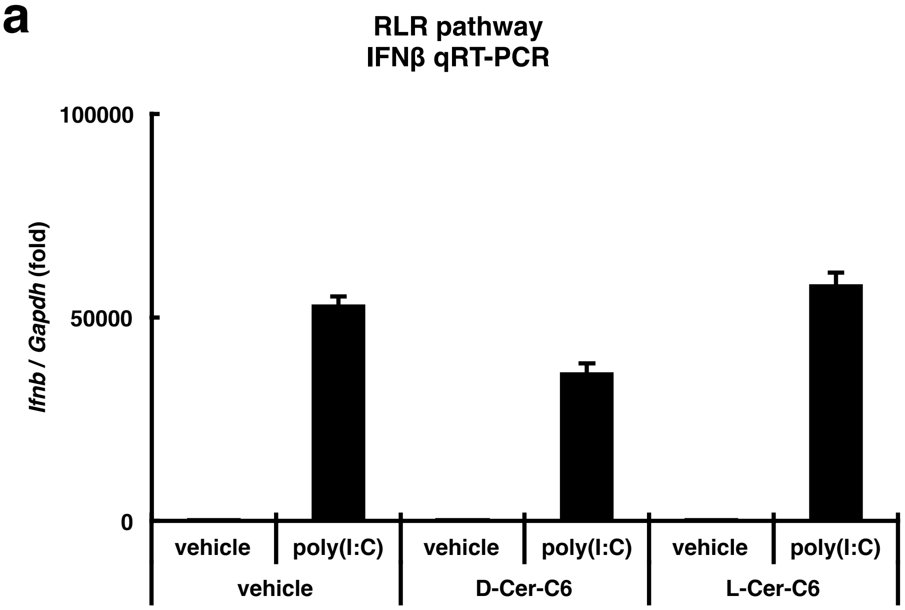

b

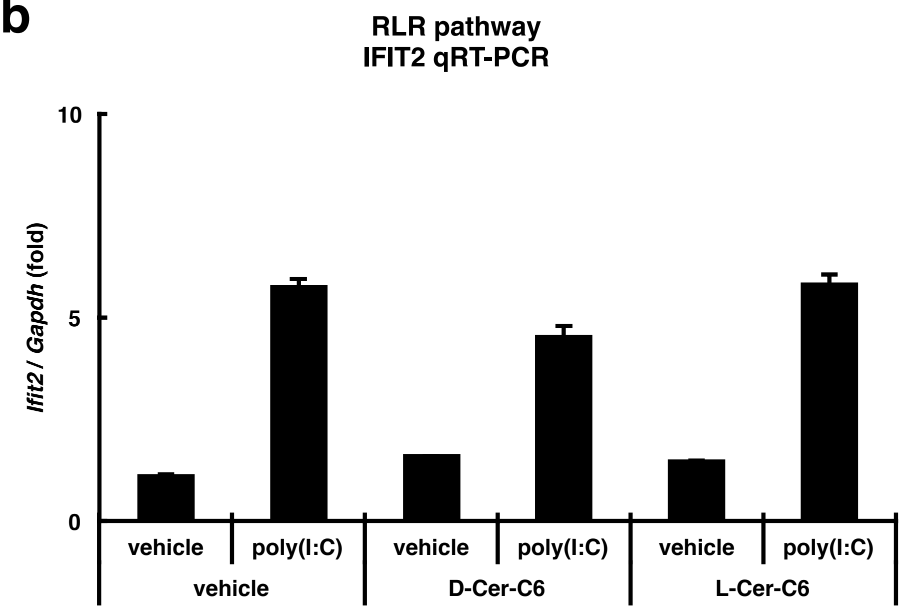

Supplementary Figure 14. **The effect of ceramide-C6 on the RLR pathways. a** and **b**, qRT–PCR of the expression of indicated genes in MEFs that were pretreated with 20 μM D-ceramide-C6 or L-ceramide-C6 for 1 h and then stimulated with the transfection of poly (I:C) for 3 h to activate the RLR pathway.

Supplementary Figure 15

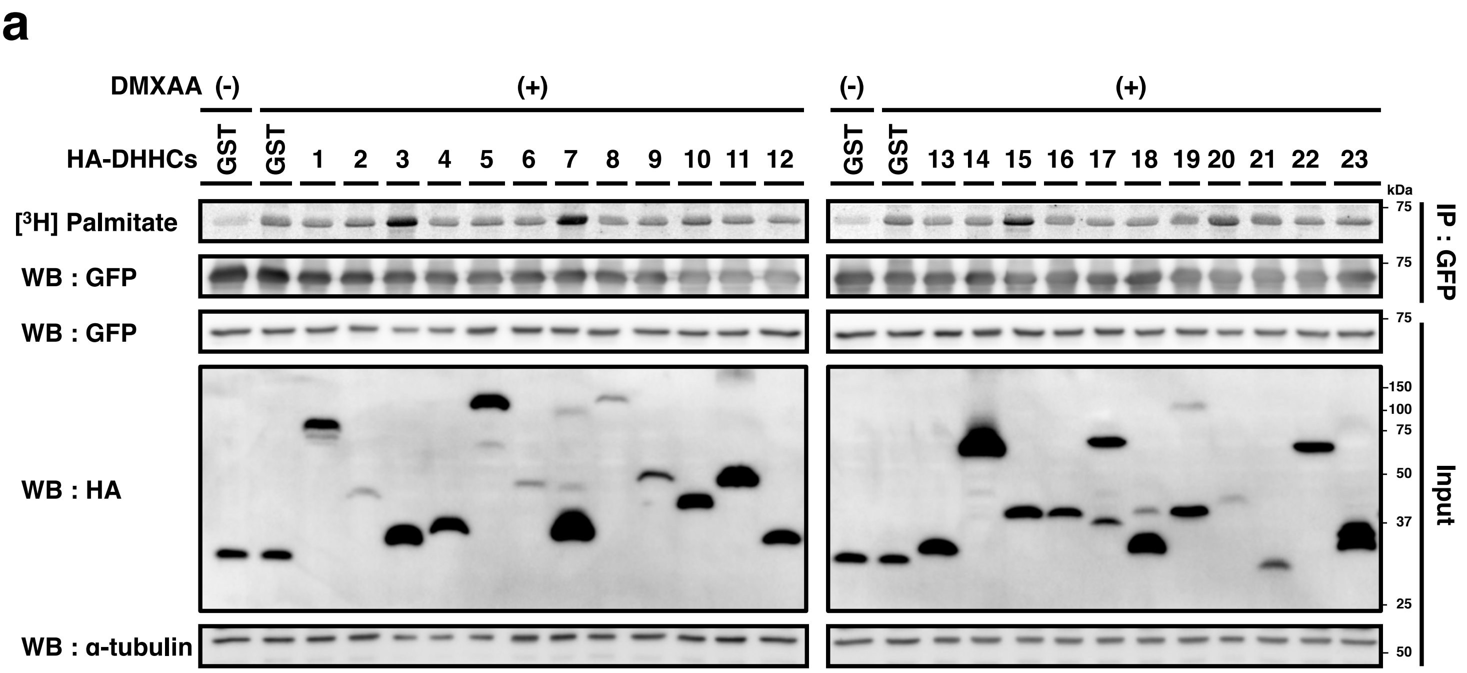

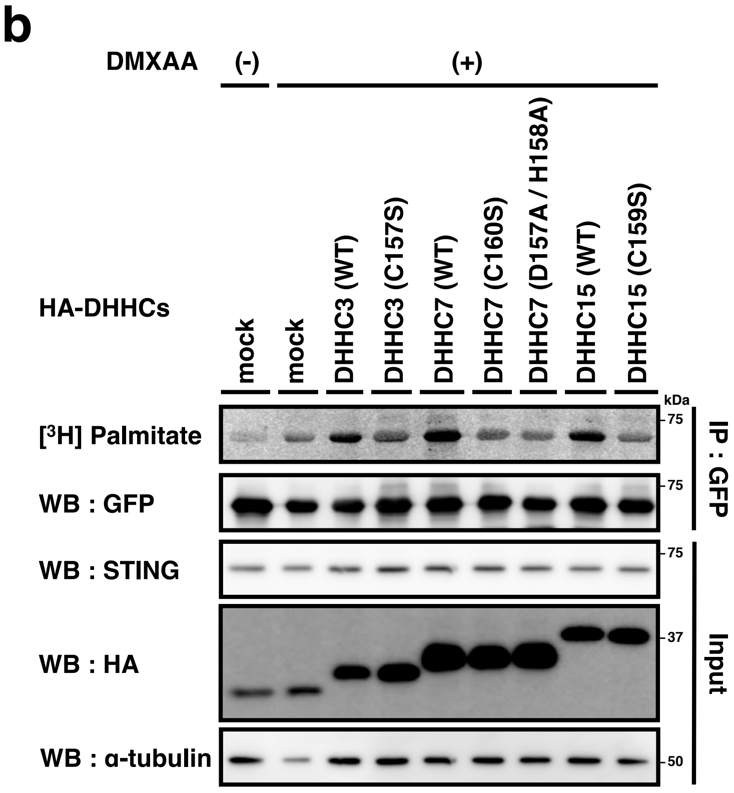

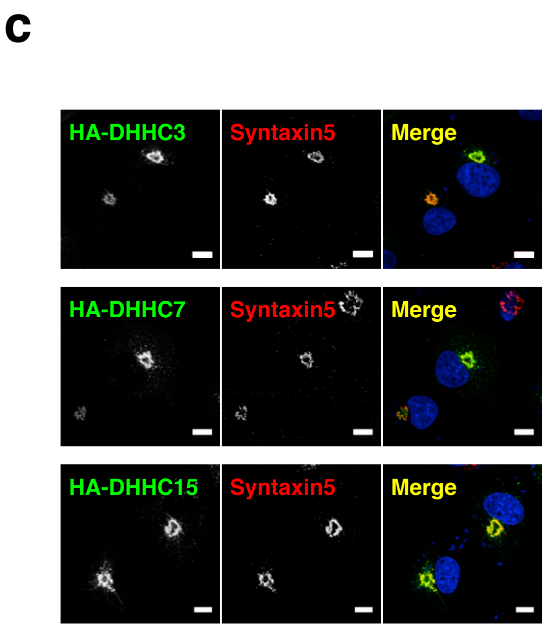

Supplementary Figure 15. Overexpression of DHHC3, DHHC7, or DHHC15 increases the palmitoylation of STING. a, emsCOS-1 cells were transfected with HA-DHHCs as indicated, metabolically labelled, and analyzed by Western blot and autoradiography. **b**, Cells were transfected with individual DHHCs (WT) or DHHC mutants (C>S or DH>AA) that lack palmitoylation activity, metabolically labelled, and analyzed by Western blot and autoradiography. **c**, COS-1 cells were transfected with HA-DHHCs, fixed, permeabilized, and co-stained for syntaxin5 (a Golgi protein). Scale bars, 10 μm.

Supplementary Figure 16

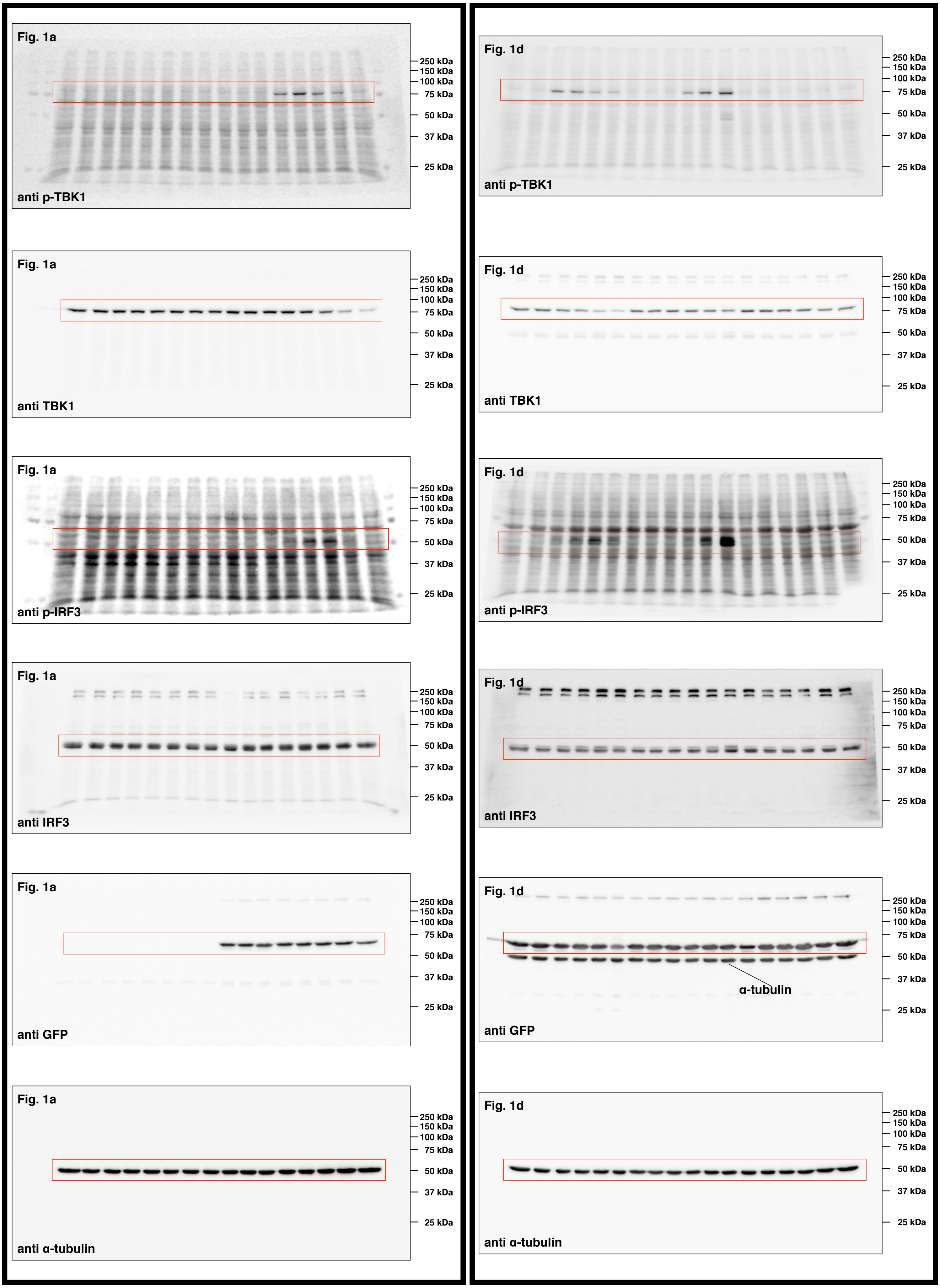

Supplementary Figure 16. Original Western blotting images used in this study.

Supplementary Figure 16 (continued)

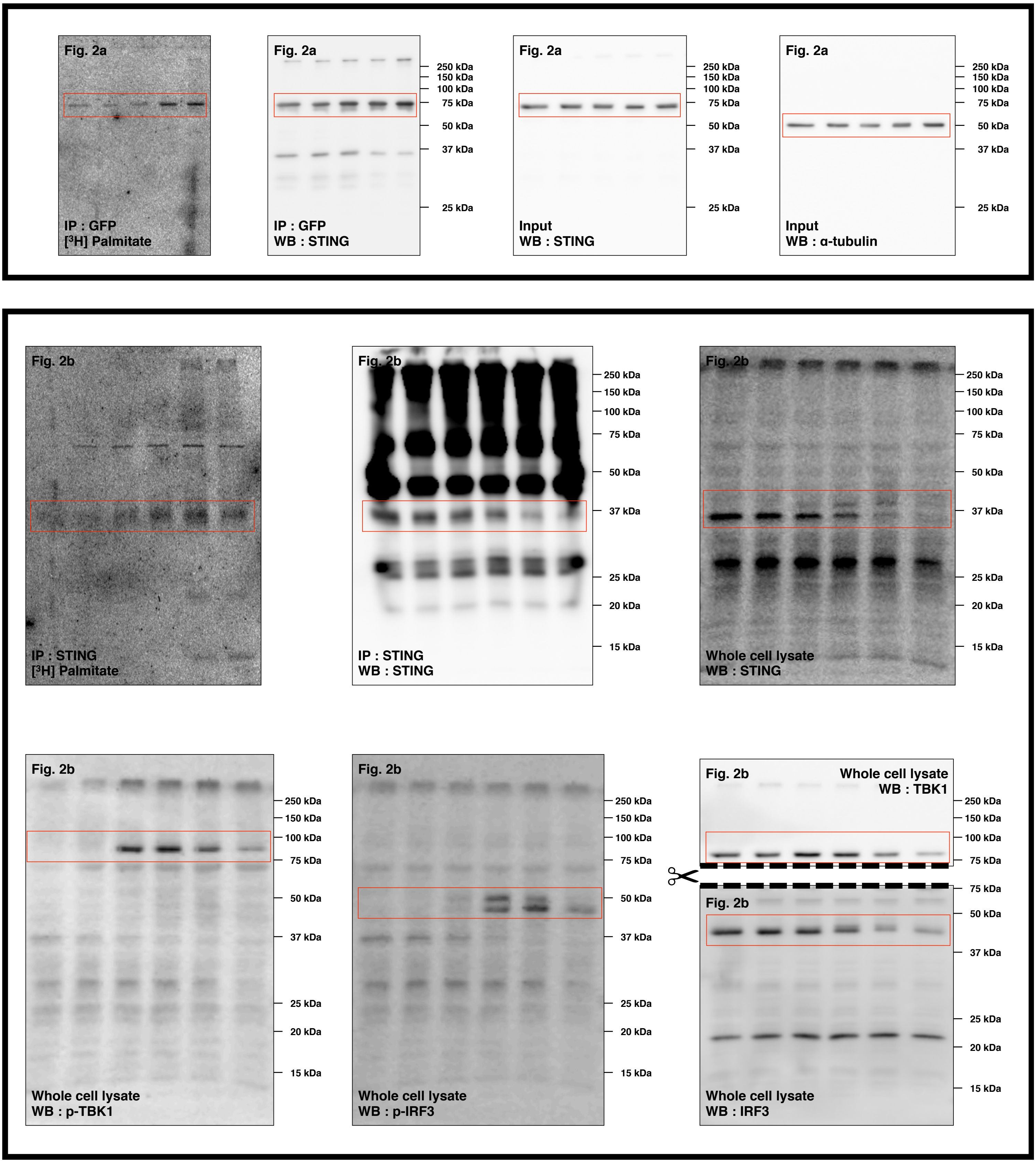

Supplementary Figure 16 (continued). Original Western blotting images used in this study.

Supplementary Figure 16 (continued)

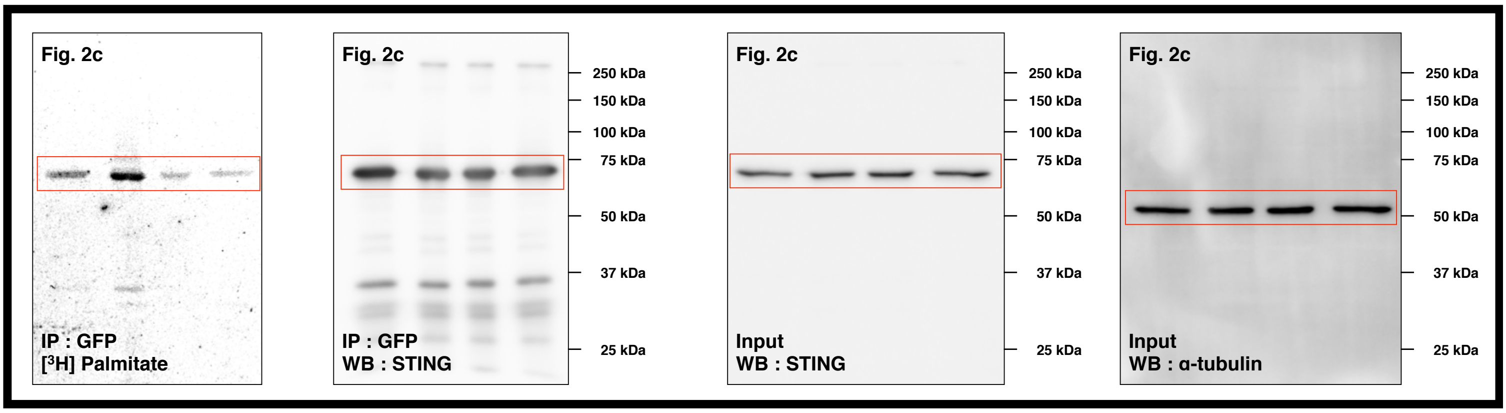

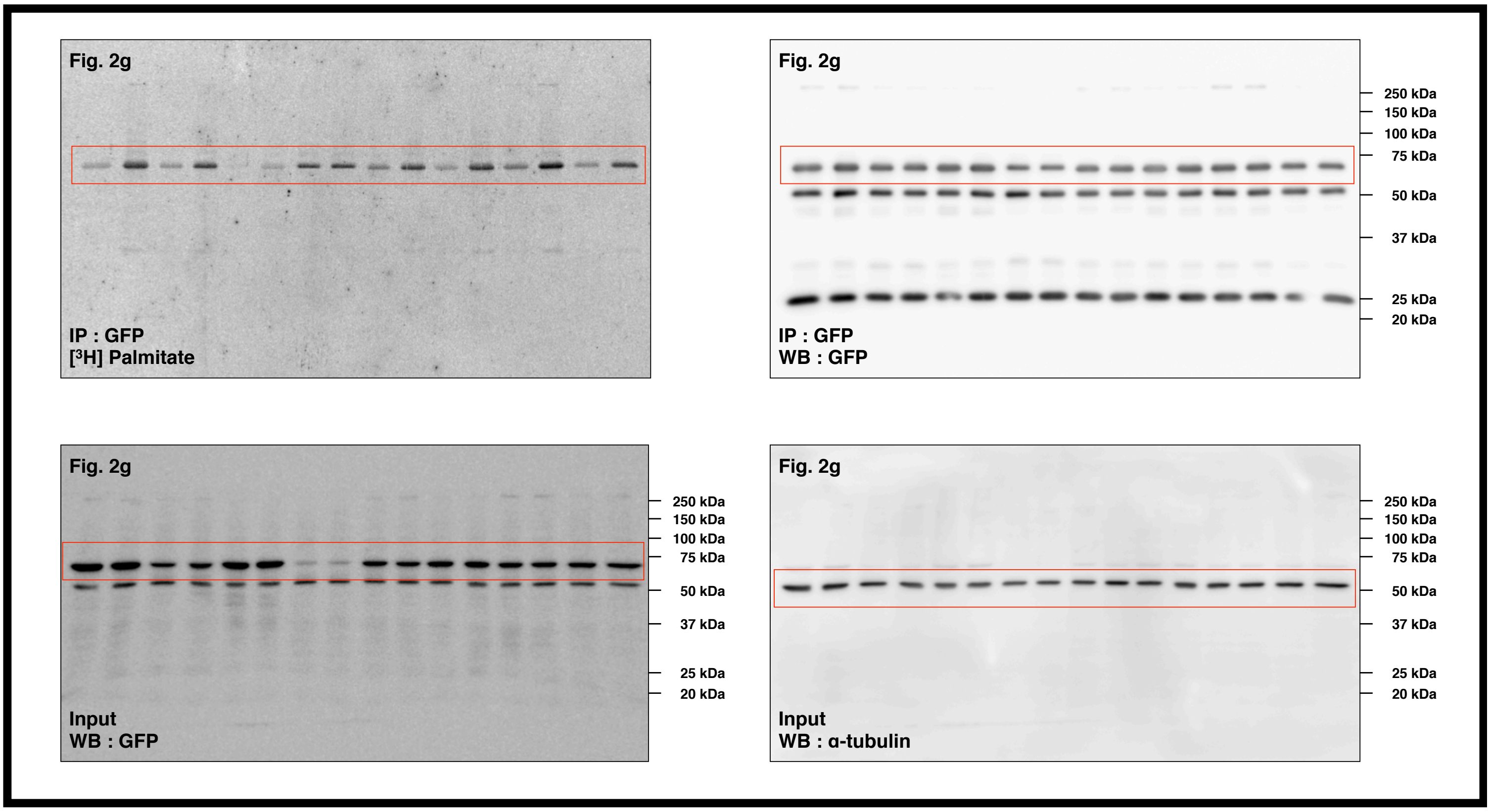

Supplementary Figure 16 (continued). Original Western blotting images used in this study.

Supplementary Figure 16 (continued)

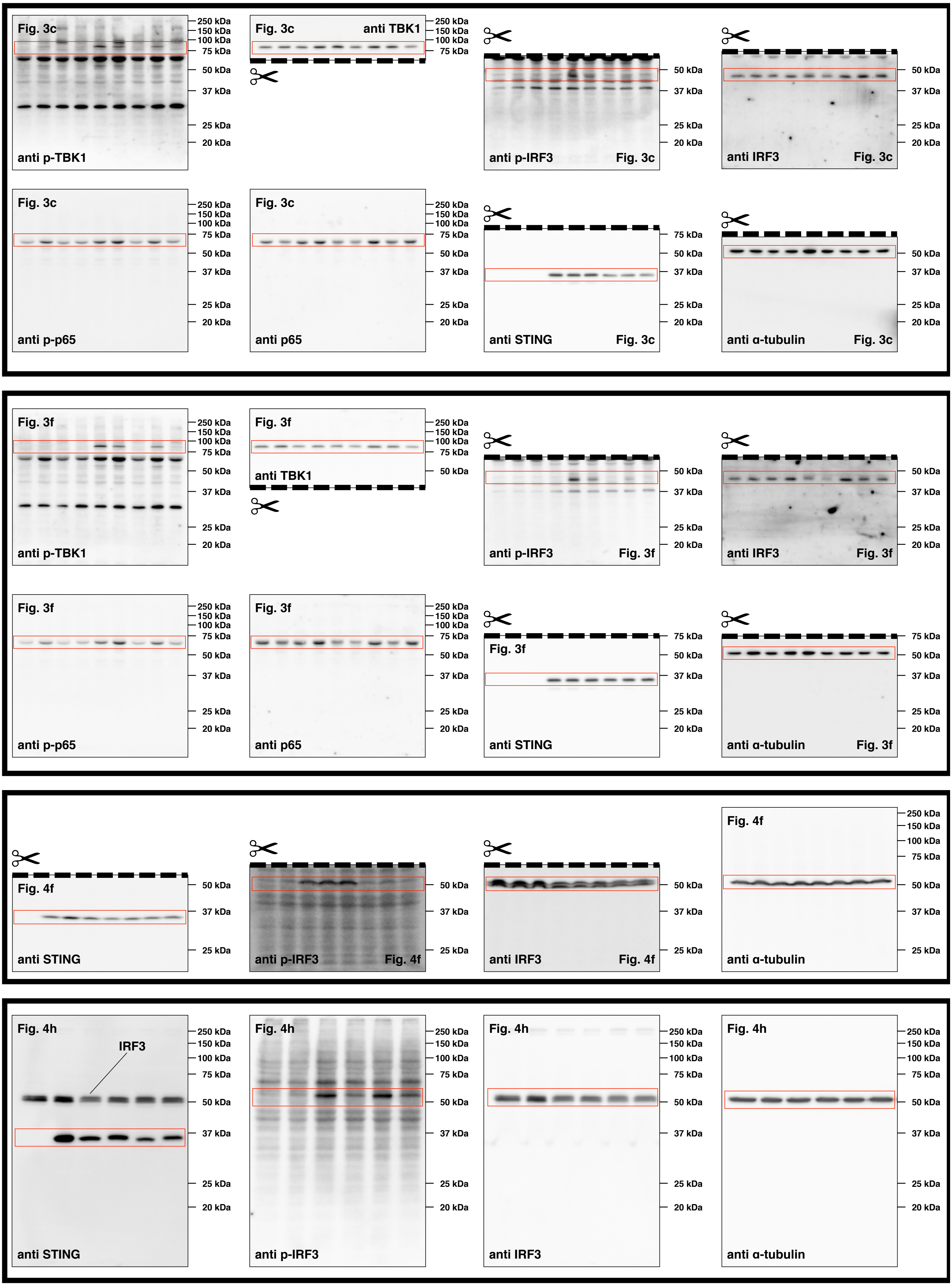

Supplementary Figure 16 (continued). Original Western blotting images used in this study.

Supplementary Figure 16 (continued)

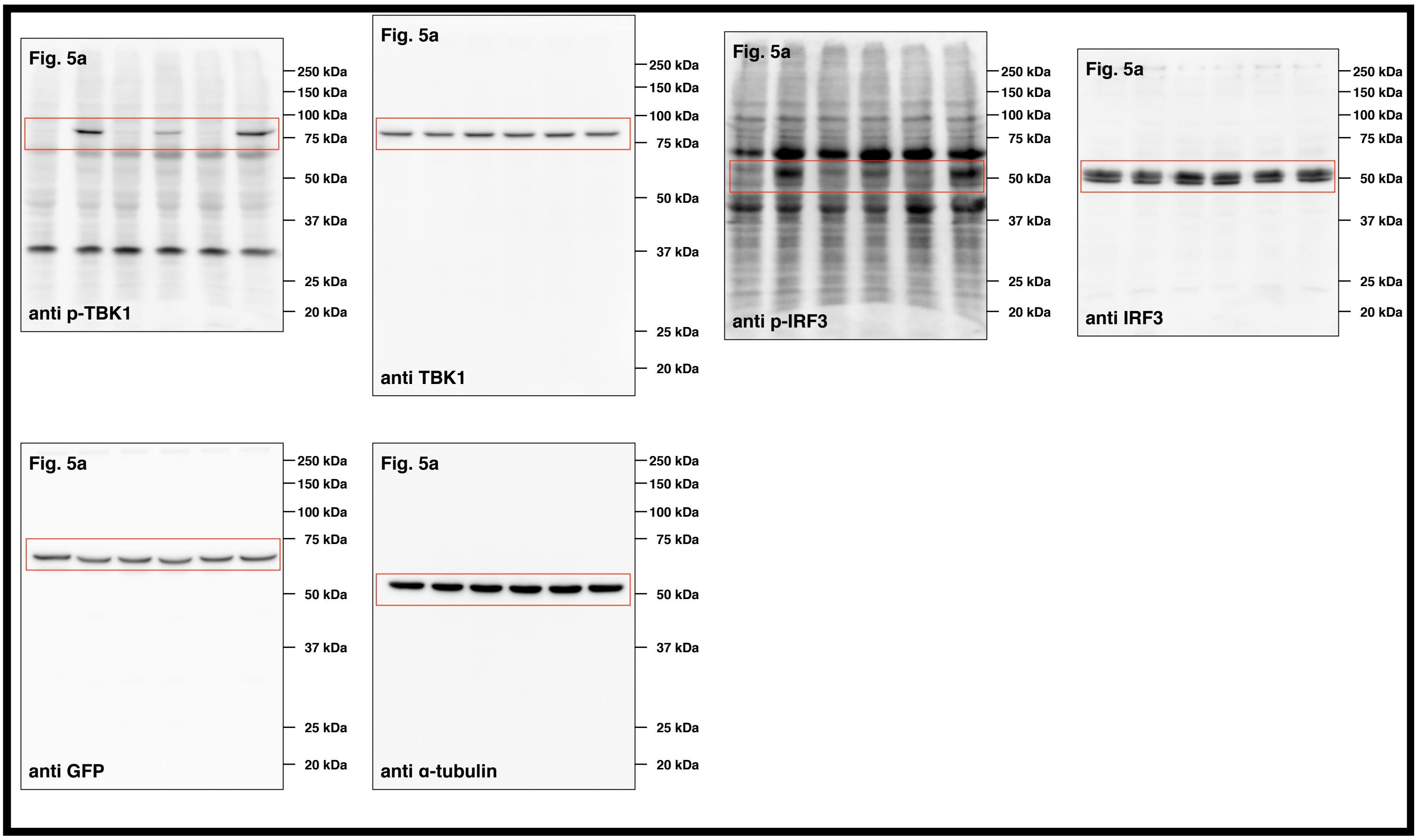

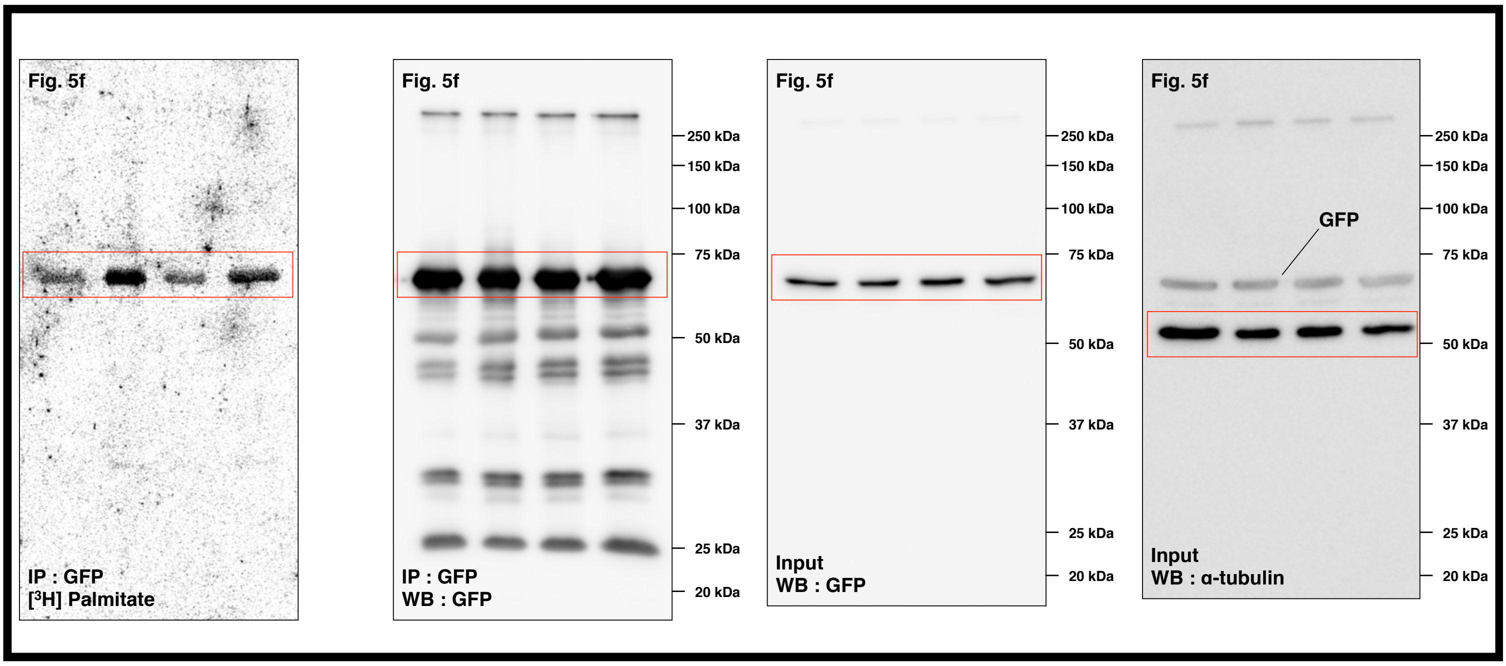

Supplementary Figure 16 (continued). Original Western blotting images used in this study.

Supplementary Figure 16 (continued)

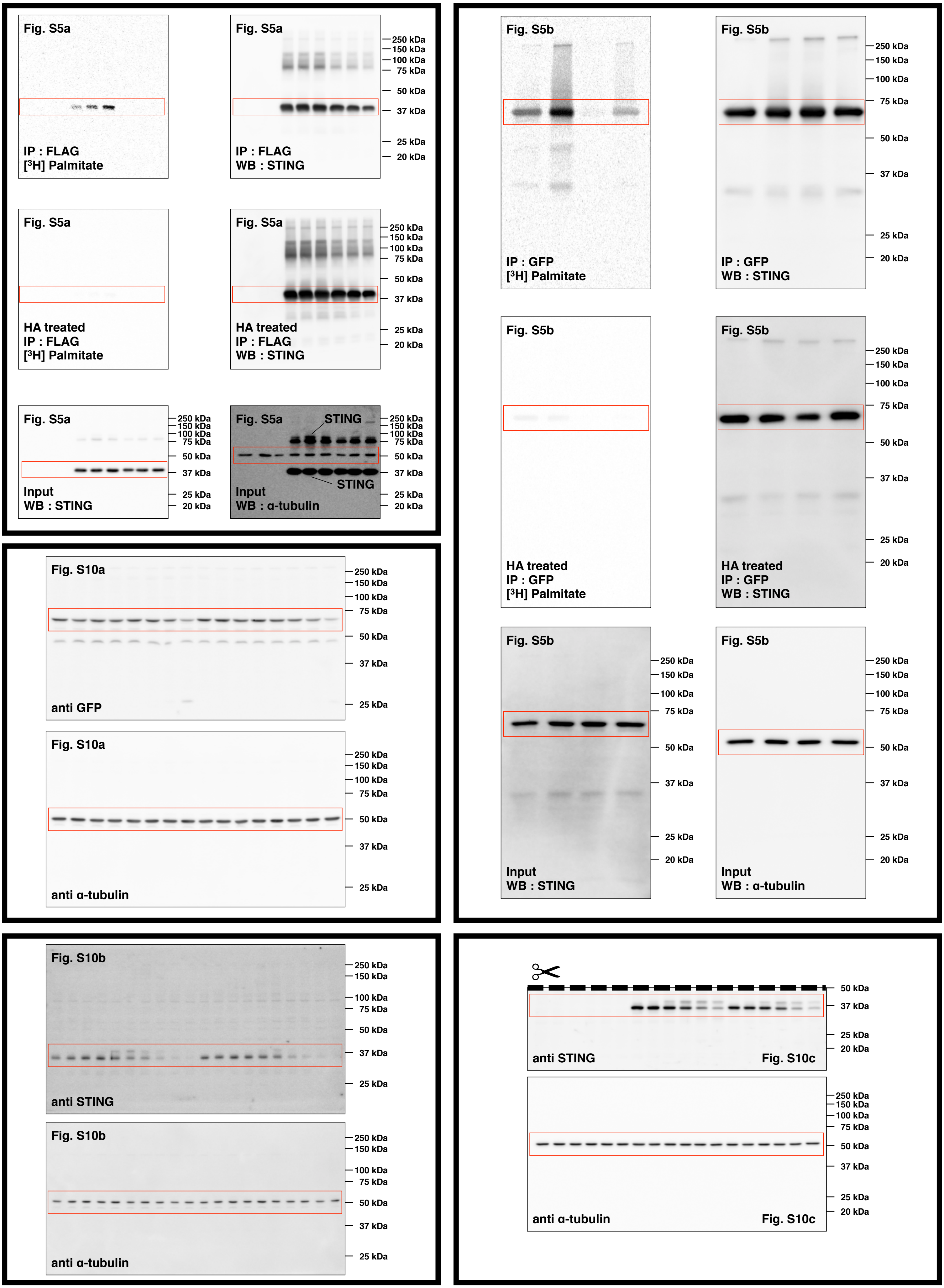

Supplementary Figure 16 (continued). Original Western blotting images used in this study.

Supplementary Figure 16 (continued)

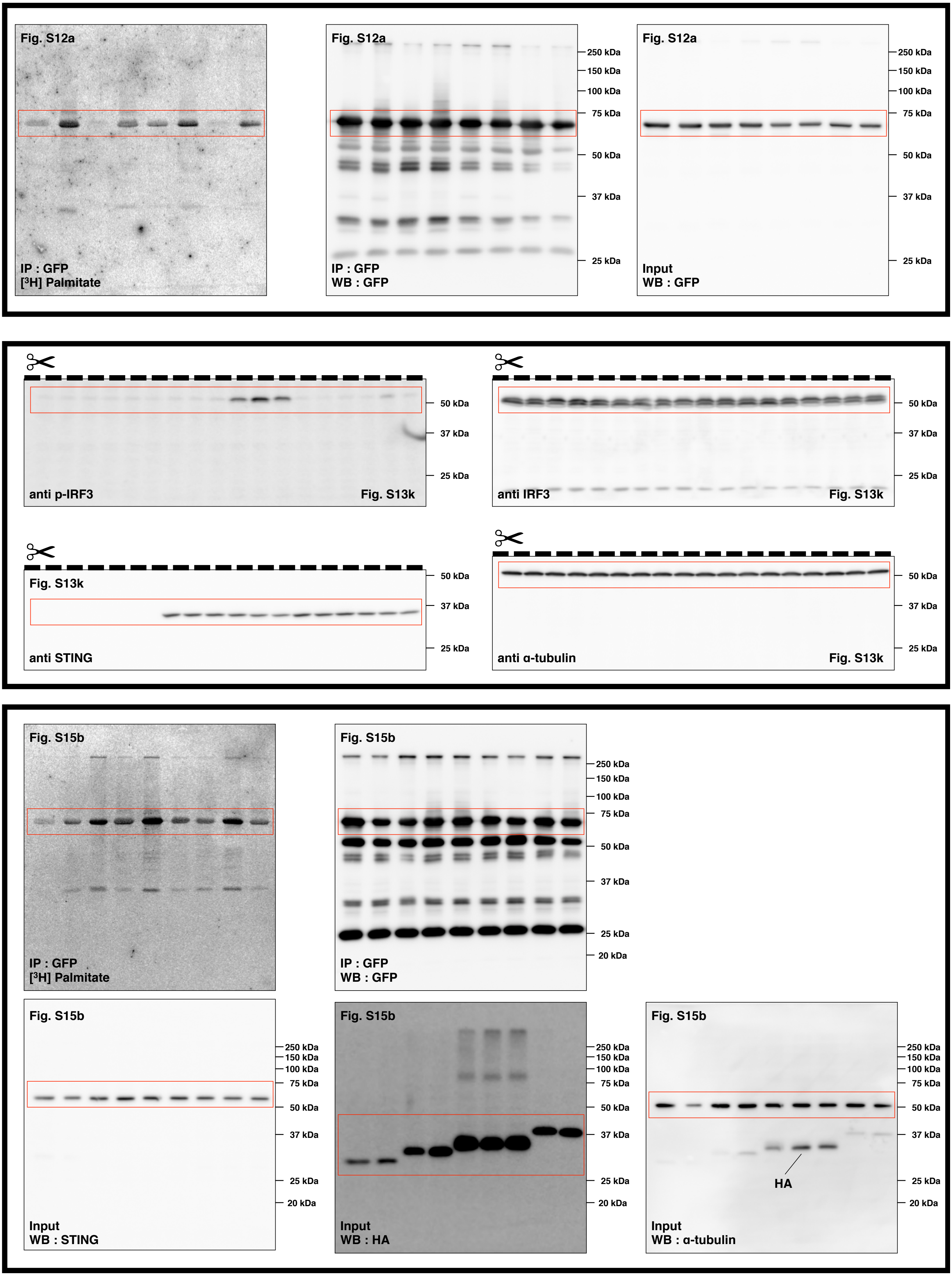

Supplementary Figure 16 (continued). Original Western blotting images used in this study.

Supplementary Figure 16 (continued)

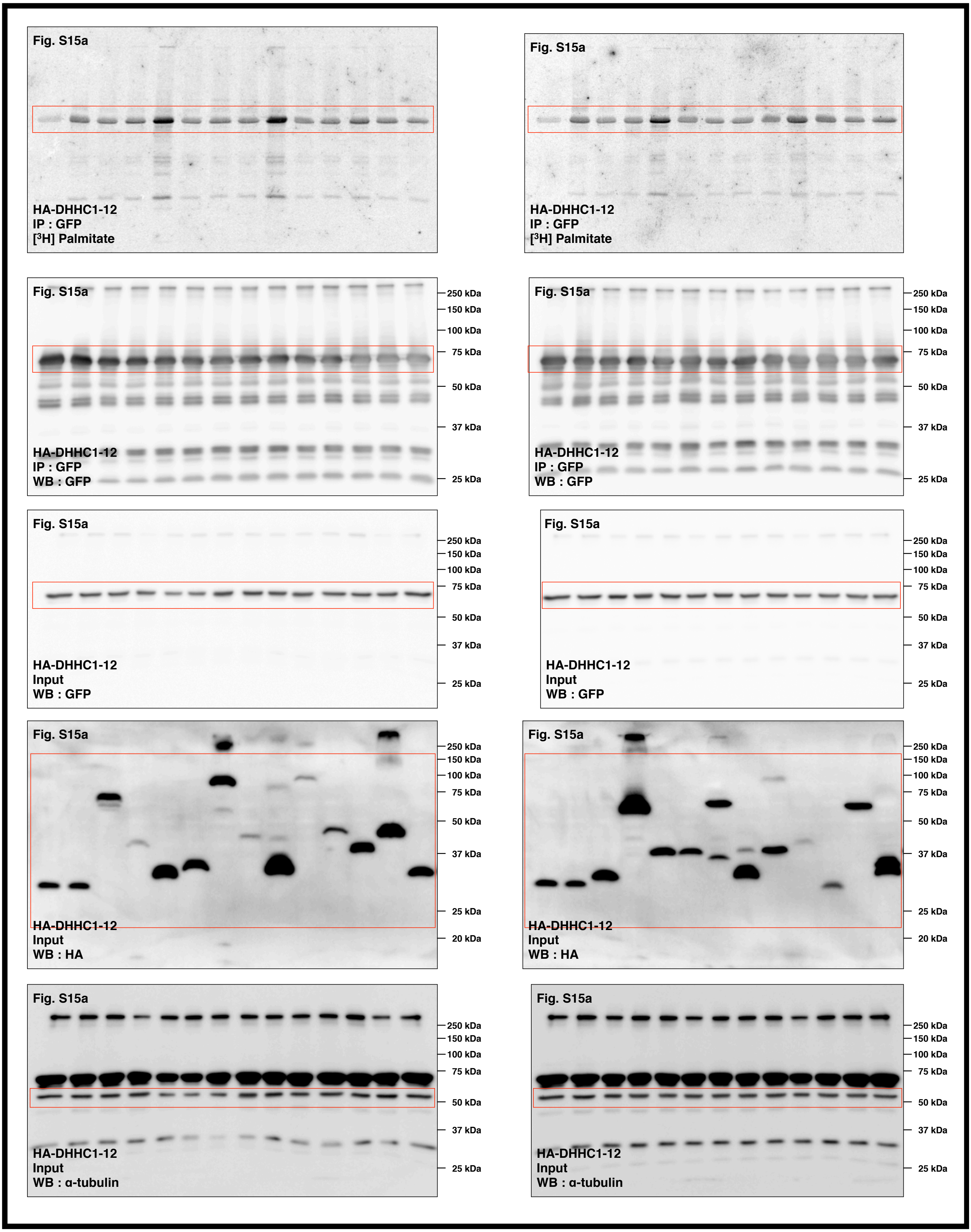

Supplementary Figure 16 (continued). Original Western blotting images used in this study.
